# Development and characterisation of acquired radioresistant breast cancer cell lines

**DOI:** 10.1186/s13014-019-1268-2

**Published:** 2019-04-15

**Authors:** Mark Gray, Arran K. Turnbull, Carol Ward, James Meehan, Carlos Martínez-Pérez, Maria Bonello, Lisa Y. Pang, Simon P. Langdon, Ian H. Kunkler, Alan Murray, David Argyle

**Affiliations:** 1 The Royal (Dick) School of Veterinary Studies and Roslin Institute, University of Edinburgh, Edinburgh, Scotland; 2 Cancer Research UK Edinburgh Centre and Division of Pathology Laboratories, Institute of Genetics and Molecular Medicine, Western General Hospital, University of Edinburgh, Edinburgh, Scotland; 3 Breast Cancer Now Edinburgh Research Team, Institute of Genetics and Molecular Medicine, Western General Hospital, University of Edinburgh, Edinburgh, Scotland; 4 Institute of Sensors, Signals and Systems, School of Engineering and Physical Sciences, Heriot-Watt University, Edinburgh, Scotland; 5 School of Engineering, Faraday Building, The King’s Buildings, University of Edinburgh, Edinburgh, Scotland

**Keywords:** Radioresistance, Breast cancer, Global gene analysis, ER and EGFR signalling, Characterisation of radioresistant cell lines

## Abstract

**Background:**

Radiotherapy plays an important role in the multimodal treatment of breast cancer. The response of a breast tumour to radiation depends not only on its innate radiosensitivity but also on tumour repopulation by cells that have developed radioresistance. Development of effective cancer treatments will require further molecular dissection of the processes that contribute to resistance.

**Methods:**

Radioresistant cell lines were established by exposing MDA-MB-231, MCF-7 and ZR-751 parental cells to increasing weekly doses of radiation. The development of radioresistance was evaluated through proliferation and colony formation assays. Phenotypic characterisation included migration and invasion assays and immunohistochemistry. Transcriptomic data were also generated for preliminary hypothesis generation involving pathway-focused analyses.

**Results:**

Proliferation and colony formation assays confirmed radioresistance. Radioresistant cells exhibited enhanced migration and invasion, with evidence of epithelial-to-mesenchymal-transition. Significantly, acquisition of radioresistance in MCF-7 and ZR-751 cell lines resulted in a loss of expression of both ERα and PgR and an increase in EGFR expression; based on transcriptomic data they changed subtype classification from their parental luminal A to HER2-overexpressing (MCF-7 RR) and normal-like (ZR-751 RR) subtypes, indicating the extent of phenotypic changes and cellular plasticity involved in this process. Radioresistant cell lines derived from ER+ cells also showed a shift from ER to EGFR signalling pathways with increased MAPK and PI3K activity.

**Conclusions:**

This is the first study to date that extensively describes the development and characterisation of three novel radioresistant breast cancer cell lines through both genetic and phenotypic analysis. More changes were identified between parental cells and their radioresistant derivatives in the ER+ (MCF-7 and ZR-751) compared with the ER- cell line (MDA-MB-231) model; however, multiple and likely interrelated mechanisms were identified that may contribute to the development of acquired resistance to radiotherapy.

**Electronic supplementary material:**

The online version of this article (10.1186/s13014-019-1268-2) contains supplementary material, which is available to authorized users.

## Background

Radiotherapy (RT) is a commonly used adjuvant treatment modality for a variety of solid cancers. Up to 83% of breast cancer patients benefit from radiotherapy either with curative or palliative intent [1]. Multiple studies, including 6 randomized control trials and subsequent meta-analyses, have shown that breast conserving surgery (BCS) followed by whole breast RT achieves survival rates and long term local control equivalent to that seen with mastectomy, with the added advantages of relatively mild toxicity and good cosmetic outcome [2–4]. However, despite the successful use of adjuvant RT in breast cancer, some patients will still develop loco-regional recurrences following the completion of a RT course. While tumour recurrence following RT can be due to residual disease or aggressive tumour biology, it can also be due to the survival of a population of cells that either have a greater intrinsic resistance to RT (e.g. hypoxic or cancer stem cells) or develop de novo resistance. These radioresistant cells can then repopulate the tumour site leading to recurrence and treatment failure.

Improved understanding of the mechanisms underlying acquired radioresistance and the development of strategies to circumvent this clinical problem are required. Previous studies have shown that multiple factors are implicated in the development of radioresistance, including deregulated signalling pathways (e.g. PI3K/AKT, NF-ƙB), oncogenic miRNA overproduction, enhanced DNA damage responses, the presence of cancer stem cells, epithelial-to-mesenchymal transition (EMT) and alterations in cancer metabolism, along with the effect of the tumour microenvironment itself (including hypoxia) [5]. Many studies focus on isolated pathways when investigating radioresistance, but it is likely that these pathways are interrelated in complex networks; for example, hypoxia can cause a more undifferentiated cellular phenotype, characterised by an increased expression of stem cell markers, which can also affect the expression of genes and pathways controlling stemness, such as Oct4, Notch and EMT [6].

In comparison to the investigation of chemoresistance, the mechanisms underlying radioresistance development are poorly understood, partly due to a lack of radioresistant model systems. The use of global approaches to investigate resistance mechanisms is gaining interest, as this allows for the study of multiple pathways simultaneously and provides an overview of complex biological systems and response to treatment [7]. In this study we developed novel in vitro radioresistant cell lines from ER positive (ER+) and ER negative (ER-) breast cancer cell lines. Parental cell lines were chosen to represent different molecular subtypes of breast cancer and included MCF-7 and ZR-751 cell lines (ER+, PgR+, HER2-), which are hormone-dependent, and the MDA-MB-231 cell line, which is triple negative (ER-, PgR-, HER2-) and consequently hormone-independent. We undertook genotypic, phenotypic and functional characterisation of our radioresistant models, enabling us to corroborate our findings at the gene, protein and functional levels. This approach allowed us not only to identify differences between parental cells and their derived RR cell lines but also between ER+ and ER- cell lines. To our knowledge, our study is the first to develop a ZR-751 RR cell line and use multicellular tumour spheroids derived from RR cells in functional assays and immunohistochemical analysis.

## Methods

### Cell culture

Cell culture reagents were obtained from Gibco Thermo Fisher Scientific (Paisley, UK), unless otherwise stated. Human breast cancer cell lines ZR-751, MCF-7 and MDA-MB-231 were cultured in Dulbecco’s modified Eagle’s medium (DMEM) supplemented with 10% foetal calf serum (FCS), 50 U ml^− 1^ penicillin and 50 mg ml^− 1^ streptomycin and incubated at 37 °C in a humidified atmosphere with 5% CO_2_. Cell lines obtained from the American Type Culture Collection (LGC Standards, Teddington, UK) were authenticated by short tandem repeat (STR) profiling performed at Health England (Porton Down, Salisbury, UK). All cell line DNA samples tested matched 9 of 9 tested core alleles in DNA from known cell line samples confirming their identity. All experiments were performed using cells, maintained at low passage number from these frozen stocks.

### Irradiation of cells and development of radioresistant cell lines

Cells were irradiated using a Faxitron cabinet X-ray system 43855D (Faxitron X-ray Corporation, IL, USA). Radioresistant cell lines (MCF-7 RR, ZR-751 RR and MDA-MB-231 RR) were developed from their respective parental cell lines (MCF-7, ZR-751 and MDA-MB-231) by weekly exposure to single fractions of radiation. An initial dose of 2 Gy was followed by weekly incremental doses of 0.5 Gy for 12 weeks. During this period cells received a total radiation dose of 57 Gy. Cells were subsequently maintained with further weekly doses of 5 Gy.

### Sulforhodamine B proliferation (SRB) assay

Cells were seeded into 96 well plates (500 cells/well) and incubated for 24 h. Cells were drug-treated or exposed to radiation and fixed between 24 and 144 h after treatment by the addition of 50 μl cold 25% trichloracetic acid (Sigma-Aldrich, UK) per well at 4 °C for 1 h. Plates were washed in H_2_O and, when dry, 50 μl SRB dye (0.4% SRB dissolved in 1% glacial acetic acid (VWR International)) was added to each well and incubated for 30 min. Plates were washed 4 times in 1% glacial acetic acid and, when dry, 150 μl of 10 mM Tris-NaOH buffer (pH 10.5) was added to each well. The plates were incubated on a shaker for 60 min. Optical density was measured at 540 nm using a Biohit BP800 spectrophotometer (Biohit Ltd., UK) and Wallac 1420 Manager program (PerkinElmer, UK). The half maximal inhibitory concentrations (IC_50_ values) were calculated using the GraphPad prism 7 package.

### Colony formation (CF) assay

Cells were seeded into 75 mm plates (1 × 10^3^ cells/plate) and incubated for 24 h before radiation treatment. Once visible colonies had formed (approximately 50 cells per colony) in the untreated control group (approximately 10–14 days post-seeding) the plates were washed twice in PBS before fixing the cells with the addition of 5 ml of 1,9-dimethyl-methylene blue zinc chloride double salt (Sigma-Aldrich, UK). After 45 min the plates were washed and allowed to air dry before colonies were counted. Analysis was performed by calculating plating efficiencies and survival fractions for control and treatment plates [8].

### Scratch (migratory) assays

Cells were seeded into 6 well plates at a density to achieve 100% confluence after 24 h. Scratch assays were performed as previously described [9]. 0.1% serum-supplemented media was added to each well. Phase contrast images of the cell monolayer were captured (Axiovert DS100, × 5 objective) during migration up to a maximum of 48 h post-scratch. At each time point the area devoid of migrating cells was calculated using FIJI software and expressed as a % of the initial scratched area.

### Formation of multicellular tumour spheroids (MTS)

A single cell suspension of each cell line from a T175 flask (approximately 15 × 10^6^ cells) was transferred to a spinner flask (Cellcontrol Spinner Flask, Integra, Switzerland) containing 100 ml of routine DMEM and placed onto a magnetic stirrer platform (Cellspin, Integra, Switzerland). MTS developed over 7 days in normal incubation conditions.

### 3D invasion assay using multicellular tumour spheroids

A single MTS was removed with 500 μl of collagen mix (ice cold 0.1% filtered acetic acid, cell matrix type 1-A (Alphalabs), 0.22 M NaOH (Sigma-Aldrich, UK), FCS and 10x DMEM (Sigma-Aldrich, UK) at concentrations of 45, 25, 10, 10 and 10%, respectively) and placed into a 24 well plate. Cultures were incubated for 1 h to allow polymerisation of collagen and 500 μl routine DMEM was then added to each well. Phase contrast images were captured (Axiovert DS100, × 5 objective) at regular intervals up to 120 h post-seeding. MTS invasion was measured using a FIJI macro developed by Matthew Pearson (IGMM Advanced Imaging Resource, University of Edinburgh) at each time point and expressed as a % of the initial MTS area.

### Assessment of the effects of radiation on pathway activation

Cells were seeded into 75 mm plates (1.0 × 10^6^ cells/plate) and incubated for 24 h. Cells were serum-starved for 2 h and exposed to 2 Gy radiation before undergoing routine lysis collection at 0, 5, 10 and 30 min post-radiation. Lysates were snap frozen on dry ice and stored at − 70 °C for western blot analysis.

### Protein isolation and detection

Whole cell lysates were prepared as previously described [10] and protein concentration was determined using a bicinchoninic acid (BCA) assay. Equal amounts of protein were separated by sodium dodecyl sulphate (SDS) polyacrylamide gel electrophoresis and transferred to Immobilon-P transfer membrane (Millipore). Ponceau S solution was used to visualise protein bands and confirm equal loading; membranes were blocked using Odyssey Blocking Buffer (LI-COR Biosciences, UK) (1:1 with PBS) for 1 h, before incubating overnight at 4 °C with primary antibodies (Table 1). Signals were detected using IRDye 800CW (Li-Cor, 926–32,210, 1:10,000) and IRDye 680LT (Li-Cor 926–68,021, 1:10,000) with a Li-Cor Odyssey Imager. Membranes required for re-probing were stripped using NewBlot PVDF Stripping Buffer (LI-COR Biosciences, UK).

**Table 1:** Primary antibodies used for western blotting (WB), immunocytochemistry (ICC) and immunohistochemistry (IHC)

Primary antibody target antigen	Antibody Details	Dilutions and applications	Antigen retrieval
Anti-α tubulin	Mouse MAb; Abcam; ab7291	1:10,000 (WB)	N/A
Anti-ERα	Mouse MAb; Dako; M7047	1:50 (WB, ICC, IHC)	Sodium citrate
Anti-EGFR	Rabbit MAb; Cell Signalling Technology; 4267	1:1000 (WB); 1:50 (ICC, IHC)	EDTA
Anti-HER2	Rabbit MAb; Cell signalling Technology; 2242	1:1000 (WB); 1:50 (ICC, IHC)	Sodium citrate
Anti-PgR (A/B)	Rabbit MAb; Cell Signalling Technology; 8757	1:1000 (WB)	N/A
Anti-PgR	Mouse Mab; DAKO; M3569	1:150 (ICC, IHC)	EDTA
Anti-AKT	Mouse MAb; Cell Signalling technology; 2920	1:1000 (WB)	N/A
Anti-Phospho AKT	Rabbit PAb; Cell Signalling technology; 9271	1:1000 (WB)	N/A
Anti-ERK	Rabbit PAb; Cell Signalling Technology; 9102	1:1000 (WB)	N/A
Anti-Phospho ERK	Mouse MAb; Cell Signalling Technology; 9106	1:1000 (WB)	N/A
Anti-ki67	Mouse MAb; DAKO; M7240	1:150 (ICC, IHC)	Sodium citrate
Anti-E-cadherin	Mouse MAb; BD Transduction; 610,182	1:50 (ICC, IHC)	Sodium citrate
Anti-N-cadherin	Mouse MAb; BD Transduction; 610,921	1:150 (ICC, IHC)	Sodium citrate
Anti-vimentin	Mouse Mab; Abcam; 8069	1:50 (ICC, IHC)	Sodium citrate
Anti-SNAIL	Rabbit PAb; Abcam; 128,530	1:250 (ICC, IHC)	Sodium citrate

### Immunohistochemistry (IHC) and immunocytochemistry (ICC)

IHC was carried out on formalin-fixed MTS. Samples were deparaffinised and rehydrated, and antigens retrieved (Table 1). Endogenous peroxidase activity was inhibited with 3% H_2_O_2_ solution (Dako, UK) for 10 min and non-specific antibody staining was blocked using Total Protein Block (Dako, UK) for 10 min. Primary antibodies were incubated for 1 h (Table 1). 1 drop of Envision labelled polymer (Dako, UK) was added for 30 min, before DAB and substrate buffer (1:50) (Dako, UK) were added to each section for 10 min.

ICC was performed on cells grown in chamber slides (2 well chamber slide, Lab-Tek II, Scientific Laboratory Supplies, UK) seeded to achieve a confluency of approximately 80% at 24 h. Cells were fixed in cold acetone (500 μl/chamber) for 10 min at 4 °C, then washed twice in PBS for 10 min. The same protocol as described for MTS IHC, from the addition of H_2_O_2_ solution, was then followed.

All slides were counterstained in haematoxylin, dehydrated and mounted with coverslips using DXP mountant (Sigma-Aldrich, UK). Slides were scanned using a NanoZoomer ER slide scanner (Hamamatsu Photonics, UK) and viewed using NanoZoomer Digital Pathology software.

### siRNA transfection

Cells were seeded into 96 or 6 well plates (500 or 2 × 10^5^ cells per well, respectively) in media without antibiotics and incubated overnight to achieve 30–50% confluence. Cells were transfected according to the manufacturer’s protocol using DharmaFECT 1 Transfection Reagent (Dharmacon) with on-target plus ERα siRNA (J-103401-12) (GE Healthcare Dharmacon) at a concentration of 25 nM.

### RNA extraction and whole-transcriptome gene expression analysis

Cells were seeded into 75 mm plates (3.0 × 10^6^ cells/plate) and incubated for 24 h. Cells were serum-starved for 2 h and were then exposed to either 0 or 2 Gy radiation. Pellets of up to 1 × 10^7^ cells were collected by trypsinisation at 0, 2 and 8 h post-radiation, snap-frozen on dry ice and stored at − 70 °C for subsequent RNA extraction. Total RNA was extracted from cells with the RNeasy Mini Kit using QIAshredder technology (UK Qiagen, Ltd). The manufacturer’s protocol for purification of total RNA from animal cells using spin technology was followed. Extracted RNA samples were quantified and assessed for the presence of contaminants using the NanoDropTM Spectrophotometer ND1000 (Thermo Fischer Scientific). Full genome expression read-counts were generated using Lexogen QuantSeq 3′ FWD sequencing technology on an Illumina flow cell which was scanned using an Illumina HiScanSQ system (Edinburgh Clinical Research Facility, University of Edinburgh). NGS reads were generated towards the poly(A) tail and read 1 directly reflects the mRNA sequence. The ZR-751 2 h, 2 Gy sample failed sequencing and was removed from further analysis. RNA integrity number (RIN) was generated for each sample to assess RNA quality (Agilent Bioanalyzer); all samples had RIN values above 9.7 (Additional file 1: Table S1). FASTQ files of raw read-count data were pre-processed using the Lexogen recommended BlueBee high-performance NGS analysis software which implemented poly(A) tail trimming and alignment to the Genome Reference Consortium Human genome build 38 reference genome using the Spliced Transcripts Alignment to a Reference (STAR) algorithm [11]. Prior to analysis, data were log2 transformed and quantile normalised in R (Bioconductor) software and packages [12]. These preliminary transcriptomic data were only used for supervised pathway-focused analyses for the purposes of hypothesis generation and each analysis was subsequently validated by lab-based experimentation. Heatmap and cluster analysis were performed using TM4 MeV (multiple experiment viewer) software [13]. Heatmap clustering was carried out using Pearson correlation with average linkage. For integration of gene expression data with public datasets correction for integration batch effects was performed in R using ComBat as described previously [14, 15]. Hierarchical clustering of parental and RR cell lines was performed using a published list of genes whose expression profile denotes the breast cancer intrinsic subtypes (basal, normal-like, Her2, luminal A and luminal B) [16]. Assignment of individual samples to intrinsic subtypes was performed using the *genefu* R package [17]. *Genefu* implements a Single Sample Predictor (SSP) algorithm which is a nearest-centroid classifier. The centroids representing the breast cancer molecular subtypes were identified through hierarchical clustering using the same intrinsic gene list that we used for cluster analysis in this study. All datasets generated and/or analysed during the current study are available in the NCBI’s Gene Expression Omnibus [18] and are accessible through GEO Series accession number GSE120798.

### Immunohistochemistry and statistical analysis

Image analysis software QuPath version 0.1.2 [19] was used to analyse ki67 and ERα target protein expression. Two-way ANOVA with Holm-Sidak’s multiple comparisons test was used to test for differences between 2 groups in CF, SRB, invasion and migration assays and western blot experiments. Unpaired (two tailed) *t-*test was used to assess differences between 2 groups in IHC analysis. *P* values < 0.05 were deemed statistically significant. Data is shown as mean ± SEM with all statistical analysis and graphs generated with GraphPad Prism 7. An overview of the samples included in each experiment (including cell line, time points, treatments and number of replicates) is provided in Additional file 2: Table S2.

## Results

### Development and confirmation of the acquisition of radioresistance in ER+ and ER- breast cancer cell lines

**Fig. 1:**
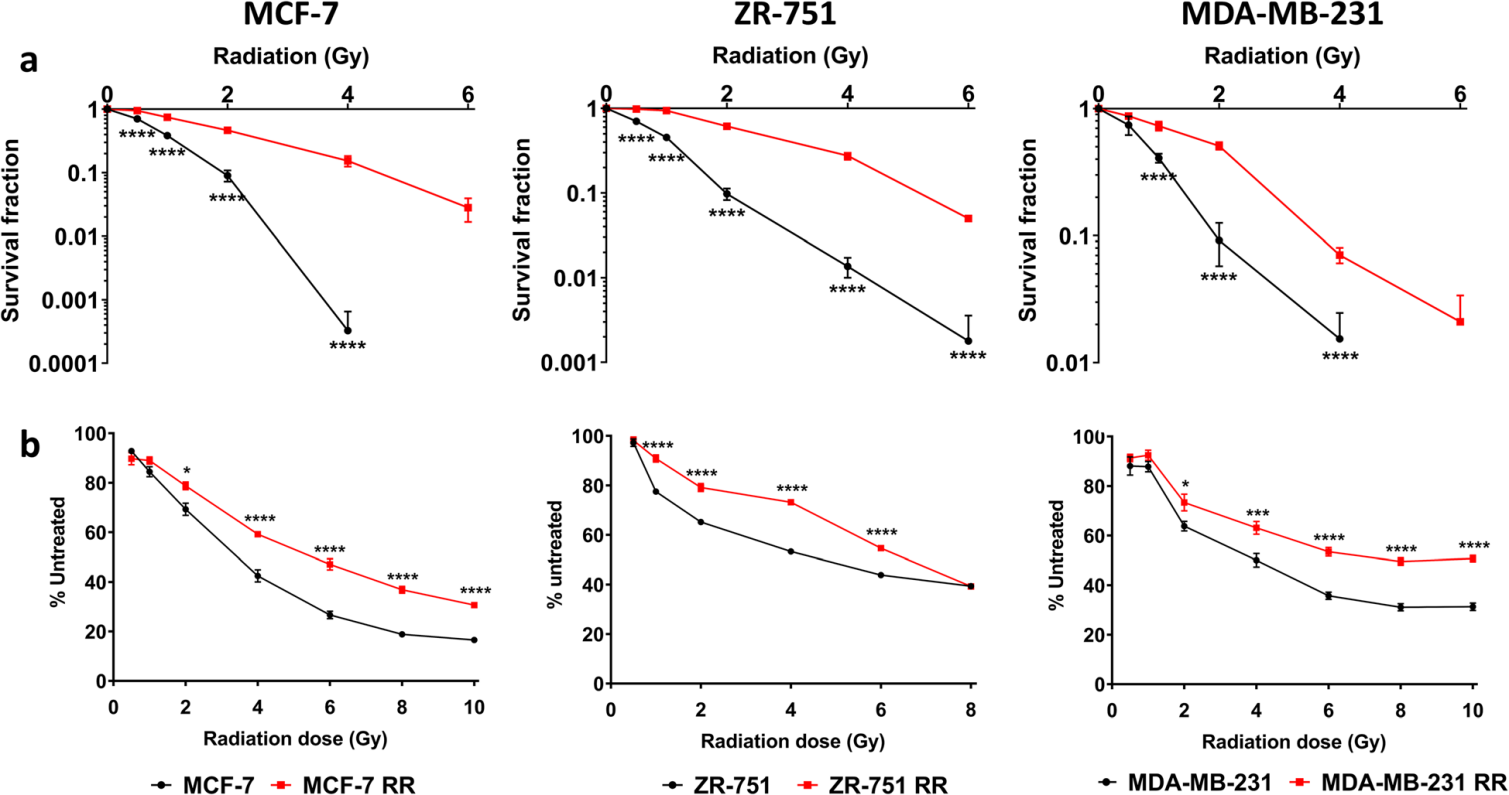
Confirmation of radioresistance using colony formation and SRB assays. **a** Colony formation assays comparing MCF-7 RR, ZR-751 RR and MDA-MB-231 RR cell lines with their respective parental cell lines. **b** SRB proliferation assays comparing MCF-7 RR, ZR-751 RR and MDA-MB-231 RR with their respective parental cell lines (2-way ANOVA with Holm-Sidak’s multiple comparisons test; data expressed as mean ± SEM, *n* = 3, ****p ≤ 0.0001; ***p ≤ 0.001; *p ≤ 0.05)

Radioresistant cell lines (MCF-7 RR, ZR-751 RR and MDA-MB-231 RR) were developed from their parental cell lines (MCF-7, ZR-751 and MDA-MB-231) by weekly exposure to single fractions of radiation, increasing by 0.5 Gy per week over a period of 12 weeks; cells were subsequently maintained by weekly doses of 5 Gy. Although maintenance radiation doses were still accompanied by cell death in all 3 RR cell lines, this was significantly less than that seen during the initial 12 week development period. Radioresistance was confirmed by both CF and SRB assays. The CF ability of all RR cell lines was significantly higher than their respective parental cell lines when exposed to a single dose of radiation up to 6 Gy (Fig. 1a). Significantly less inhibition of proliferation was also seen in the RR cell lines compared to their parental cell lines when exposed to a single dose of radiation up to 10 Gy (Fig. 1b). The IC_50_ (the dose of radiation required to reduce cell number by 50%) values were higher in the RR cell lines when compared to their parental cells (Table 2). MCF-7 RR and MCF-7 RR cells which had not received radiation for 6 months (MCF-7 rr) were similarly radioresistant (Additional file 3: Figure S1), suggesting the longevity of the changes involved in the acquisition of this phenotype.

**Table 2:** Table of IC_50_ values for each parental and RR cell line up to 144 h post-exposure to radiation. If no value is recorded this indicates that a reduction in proliferation by 50% at that time point had not occurred; MCF-7 and MDA-MB-231 cell lines were evaluated up to 120 h post radiation whereas ZR-751 cell line was evaluated up to 144 h post radiation

Time Post Radiation (h)	MCF-7	MCF-7 RR	ZR-75-1	ZR-75-1 RR	MDA-MB-231	MDA-MB-231 RR
24	–	–	–	–	–	–
48	–	–	–	–	–	–
72	4.98	9.64	–	–	–	–
96	3.27	5.36	–	–	3.53	9.71
120	3.74	5.22	–	–	3.13	7.11
144	N/A	N/A	4.38	6.91	N/A	N/A

### Radioresistant cell lines have concurrent lower expression of cell cycle associated genes and modified basal proliferation rates relative to their parental cells

**Fig. 2:**
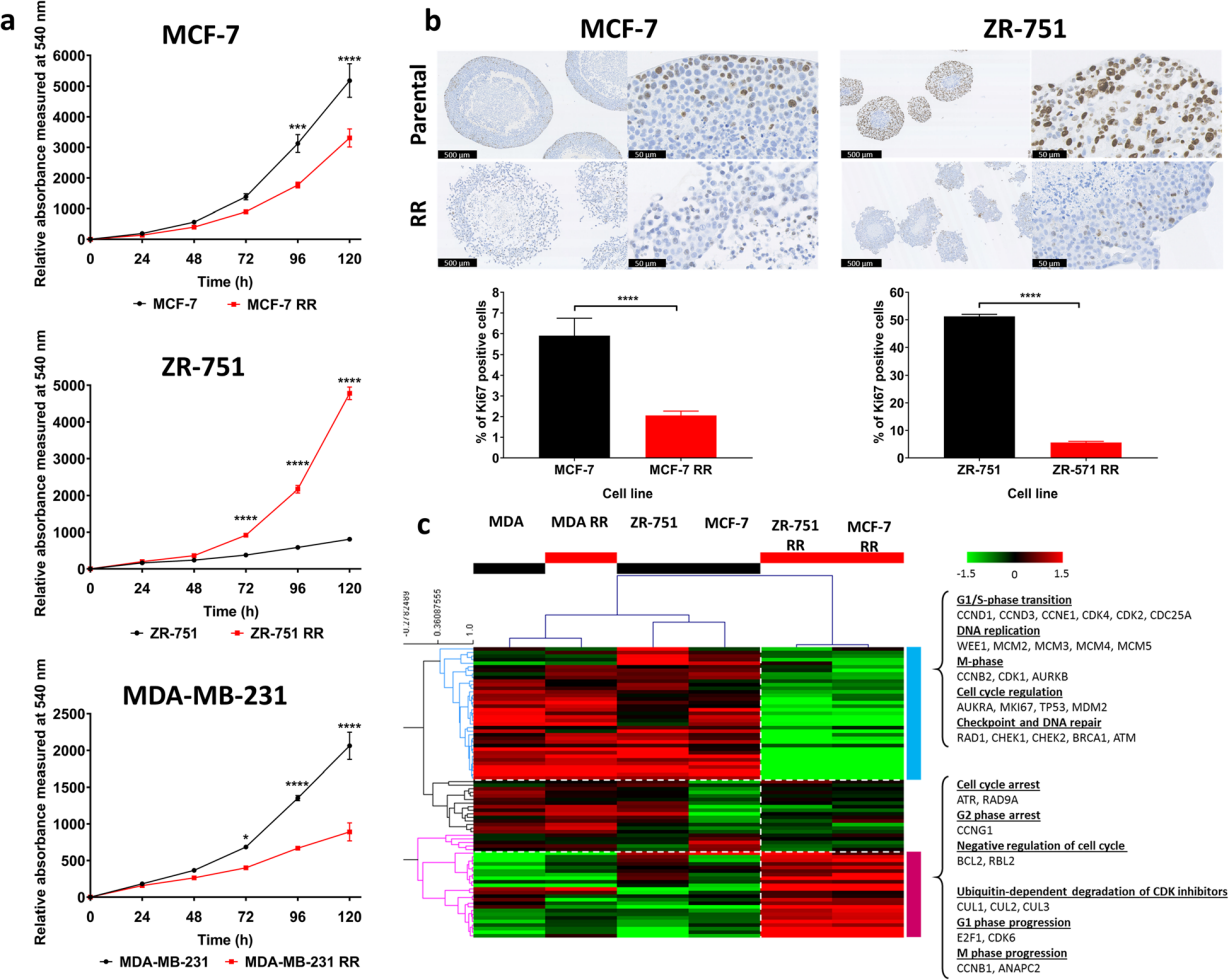
Radioresistant cell lines have modified basal proliferation rates relative to their parental cells. **a** SRB assays showing differences in proliferation rates between parental and RR cell lines grown in 2D cultures (2-way ANOVA with Holm-Sidak’s multiple comparisons test). **b** IHC of MTS stained for ki67 using MCF-7 and ZR-751 parental and RR cell lines with quantitative analysis of the % of positively stained cells (unpaired, two tailed *t-*test) (data expressed as mean ± SEM, *n* = 3, ****p ≤ 0.0001; ***p ≤ 0.001; *p ≤ 0.05). **c** Heatmap showing log2 mean-centered gene expression profiles of proliferation genes in parental and RR cell lines taken from the KEGG database cell cycle pathway; red = higher expression, green = lower expression. Heatmap clustering was carried out using Pearson correlation with average linkage

SRB assays were used initially to assess proliferation rates in 2D cultures (Fig. 2a). Results showed lower rates of proliferation in the MCF-7 RR and MDA-MB-231 RR and higher rates in the ZR-751 RR cell lines in comparison to their respective parental cell lines. Further investigation of proliferation was performed through 3D assays using IHC staining for the proliferation marker Ki67 in MCF-7 and ZR-751 parental and RR MTS (Fig. 2b) (MDA-MB-231 cell lines failed to develop MTS which could withstand IHC processing). IHC with quantitative analysis showed a lower percentage of positively stained ki67 cells in the RR MTS suggesting lower basal proliferation rates than their parental cell lines. Using gene expression data from 2D cultures, MCF-7 RR and ZR-751 RR cell lines were characterised by lower expression of genes involved in DNA replication and repair and those related to G1/S-phase transition and regulation of cell cycle, including AURKA and TP53, along with the higher expression of cell cycle arrest genes. Fewer changes in these genes were identified in the triple negative MDA-MB-231 parental and RR cell lines, both of which clustered next to each other and the MCF-7 and ZR-751 parental cell lines (Fig. 2c).

### Radioresistant cell lines have increased invasion and migration potential

**Fig. 3:**
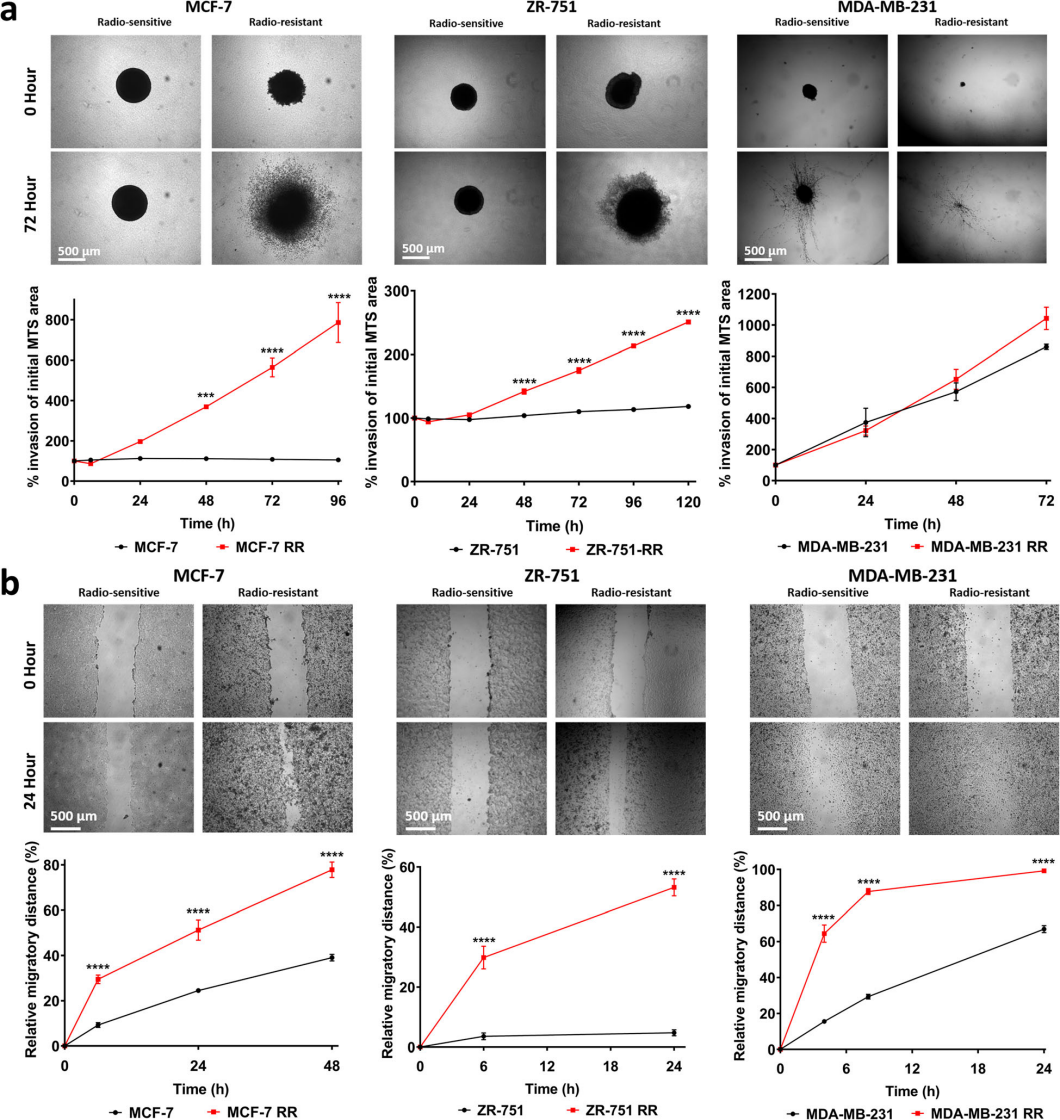
Radioresistant cell lines have increased invasion and migration potential. **a** 3D MTS invasion assays comparing MCF-7 RR, ZR-751 RR and MDA-MB-231 RR MTS with their respective parental MTS. MTS were placed in collagen and invasion was assessed up to 120 h post-seeding. Area of MTS at each time point was calculated and expressed as a % of initial MTS area at day 0. **b** 2D migration scratch assays comparing MCF-7 RR, ZR-751 RR and MDA-MB-231 RR cell lines with their respective parental cells. Relative migratory distance was calculated at each time point and expressed as a % area devoid of cells based on the initial scratched area at day 0 (2-way ANOVA with Holm-Sidak’s multiple comparisons test; data expressed as mean ± SEM, *n* = 3, ****p ≤ 0.0001; ***p ≤ 0.001; **p ≤ 0.01)

Epithelial-to-mesenchymal transition (EMT) is considered a normal feature of embryogenesis involved with cellular movement and morphogenesis during embryonic development [20] and may be an inherent property of normal basal stem cells in the breast [21]. However, the process can also be involved in tumour development with the conversion of early stage non-invasive tumours into invasive malignancies [22]. Acquisition of an EMT phenotype is associated with increased migration and invasion potential. Here, using a 3D invasion assay, we showed that both MCF-7 RR and ZR-751 RR have increased invasive potential compared to parental cells (which demonstrated little or no invasive capabilities); although the MDA-MB-231 RR cell line showed a slight increase in invasiveness at 72 h, this was not statistically significant (Fig. 3a). Similarly, we assayed for migration potential using a 2D scratch assay and showed that all 3 RR cell lines had significantly enhanced migratory ability compared to their parental cells (Fig. 3b).

**Fig. 4:**
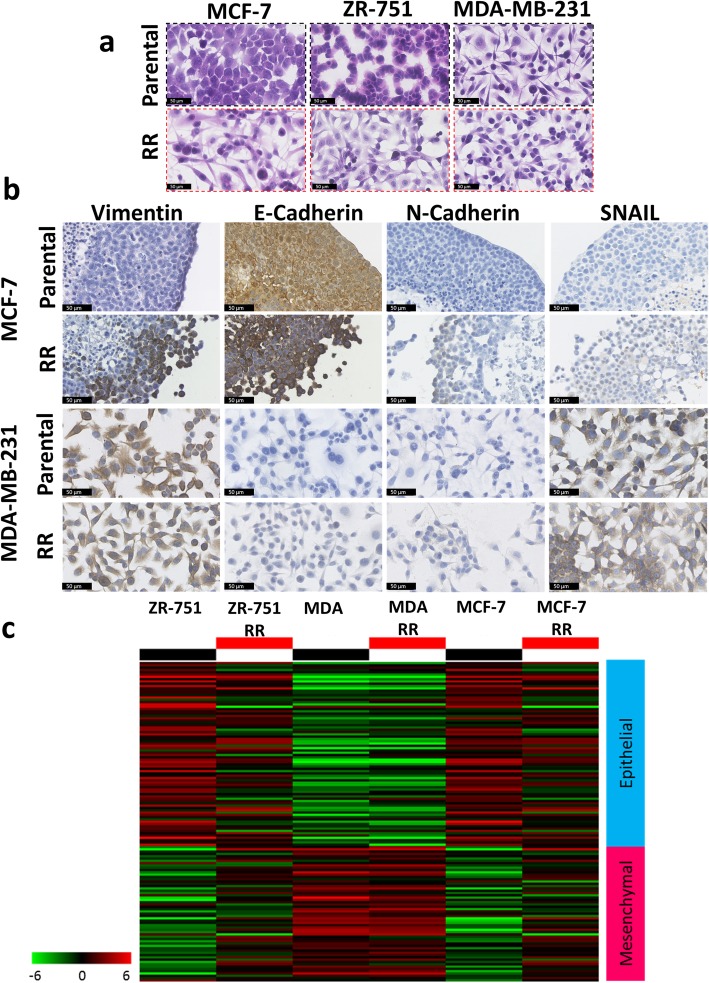
MCF-7 RR and ZR-751 RR cell lines show increased expression of vimentin, N-cadherin and SNAIL with the partial down regulation of E-cadherin. **a** H&E staining of parental and RR cell lines detailing their morphological differences. **b** IHC staining of EMT markers (vimentin, N-cadherin, SNAIL and E-cadherin) in MCF-7 and MDA-MB-231 parental and RR MTS and cells. **c** Heatmap showing log2 mean-centered gene expression profiles in respect of a published cancer cell EMT-signature [23]; red = higher expression, green = lower. The gene list and the order in which they appear in the heatmap are shown in Additional file 6: Table S3

Parental ER+ cell lines exhibited a typical epithelial-like morphology, consisting of tightly packed cells forming cobblestone-like monolayers, with the cells consisting of a large nucleus and small amount of cytoplasm. By 12 weeks post-radiation, morphological changes were observed in their RR derivatives (Fig. 4a). Individual cells or those growing in small clusters gained a more spindle-shaped morphology with a larger amount of cytoplasm, with the contact between cells now via focal points rather than cell clusters. The MDA-MB-231 cell line already exhibited a mesenchymal-like phenotype, and phenotypic changes in its RR derivate were not obvious.

The observed change in cell morphology with the acquisition of radioresistance is consistent with cells undergoing EMT. We therefore investigated the expression levels of EMT markers using IHC for the MCF-7 and ZR-751 parental and RR cell lines and identified increased expression of vimentin, N-cadherin and SNAIL, along with the partial down regulation of E-cadherin in the RR cell lines. The MDA-MB-231 cell line was characterised by high vimentin along with low N-cadherin and E-cadherin expression; no differences between parental and its RR derivative were identified (Fig. 4b and Additional file 4: Figure S2 and Additional file 5: Figure S3). These results were recapitulated using gene expression analysis from a published EMT signature (Fig. 4c) [23]. This study combined bioinformatic expression data analysis from both The Cancer Genome Atlas and Cancer Cell Line Encyclopaedia databases of seven tumour types which identified a pan-cancer EMT-associated gene expression signature. The lists of genes used have been provided in Additional file 6: Table S3. In our analysis, the MCF-7 and ZR-751 parental cell lines had a pattern of expression consistent with an epithelial genotype, whereas the MDA-MB-231 (both parental and RR) cells had higher expression of the mesenchymal cluster of genes. The MCF-7 RR and ZR-751 RR cell lines had a mixed pattern of expression with relatively high expression of both epithelial and mesenchymal genes, suggesting a transition from an epithelial towards a mesenchymal expression profile (Fig. 4c).

### WNT signalling is increased in ER+ derived radioresistant cell lines

**Fig. 5:**
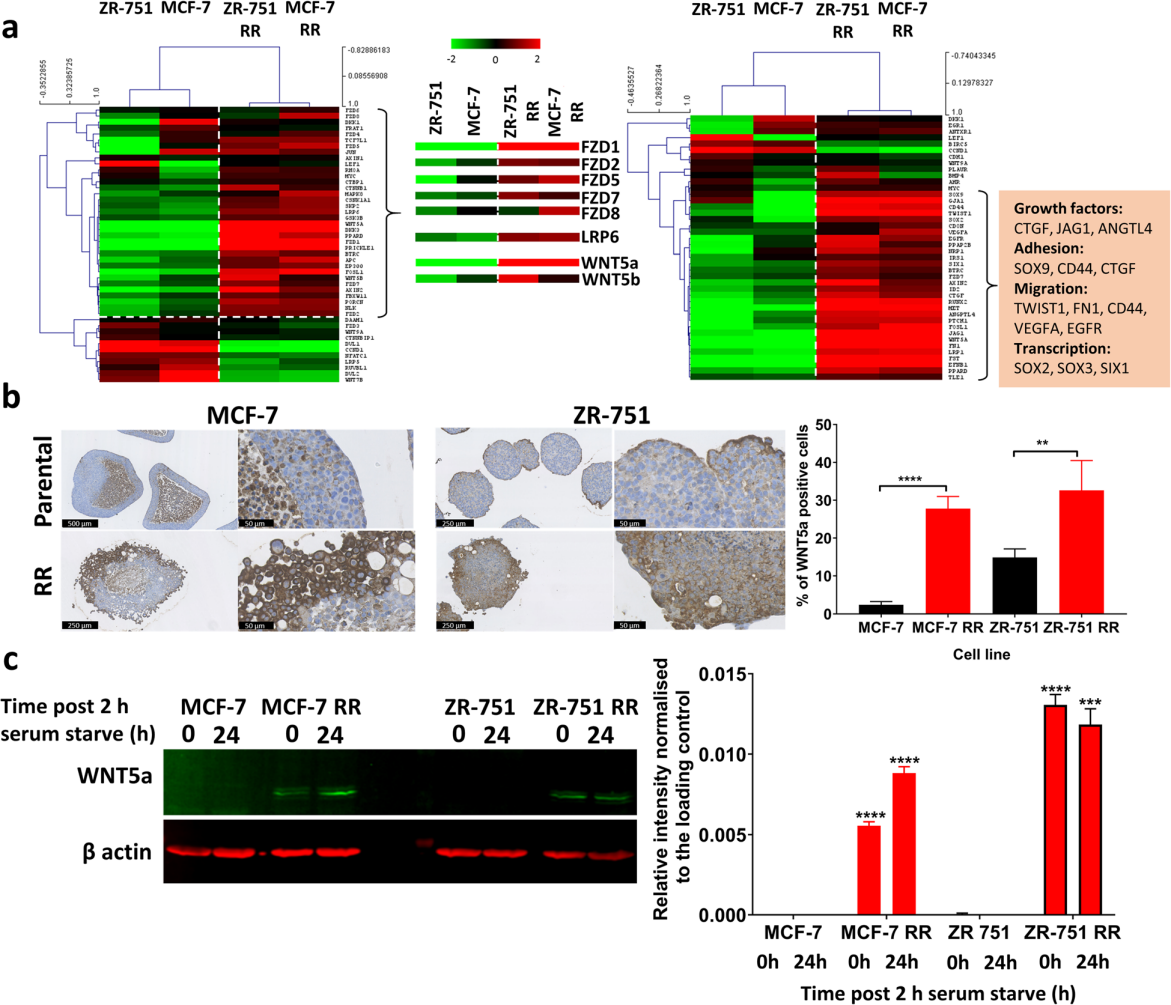
WNT signalling is increased in ER+ derived radioresistant cell lines. **a** Heatmap showing log2 mean-centered gene expression profiles between parental and RR cell lines in respect of the WNT signalling pathway (left heatmap) with selected WNT and FRIZZLED genes shown (center) and WNT target genes (right), taken from the KEGG pathway database [25]; red = higher expression, green = lower expression. The gene list and the order in which they appear in the heatmap are shown in Additional file 6: Table S3. Heatmap clustering was carried out using Pearson correlation with average linkage. **b** IHC of WNT5a expression using MCF-7 and ZR-751 parental and RR MTS. **c** Western blot analysis showing the levels of WNT5a in untreated MCF-7and ZR-751 cell lines in comparison to their RR derivatives (samples were obtained at 0 and 24 h after a 2 h serum starve), (unpaired, two tailed *t-*test; data expressed as mean ± SEM, *n* = 3, ****p ≤ 0.0001; ***p ≤ 0.001, ** *p* ≤ 0.01)

Analysis of transcriptomic data from ER+ parental and RR cell lines in respect of the WNT signalling pathway was investigated due to its reputed role in EMT and radioresistance [24]. The WNT pathway was investigated using panels of WNT signalling pathway members and down-stream targets, taken from the KEGG database [25]. The lists of genes used have been given in Additional file 6: Table S3. In this analysis the MCF7 RR and ZR-751 RR cell lines had a pattern of expression consistent with WNT pathway activation enriched for genes including FRIZZLED family members 1/2/5/7, WNT5a and WNT5b (Fig. 5a). IHC using MCF-7 and ZR-751 parental and RR MTS showed increased expression of WNT5a in the radioresistant cell lines which was predominantly expressed in the cells located in the outer proliferating layer of the MTS (Fig. 5b). Further validation through western blot analysis of serum starved whole cell lysates (in accordance with gene analysis experiments) also showed increased WNT5a expression in the radioresistant cell lines at 0 and 24 h post-serum starvation (Fig. 5c).

### Radioresistant MCF-7 and ZR-751 cell lines show loss of ERα and PgR expression and gain in EGFR expression

**Fig. 6:**
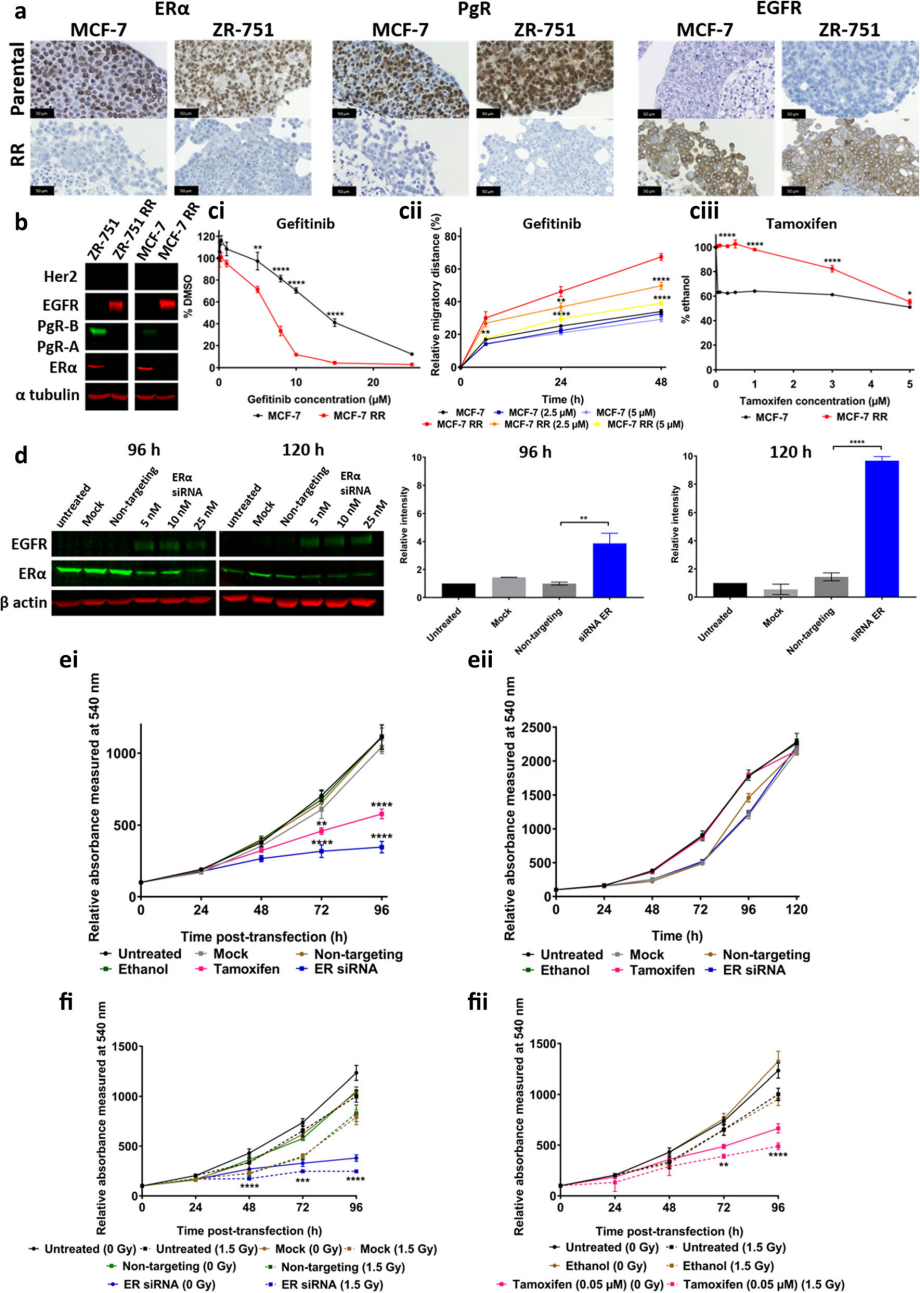
Radioresistant MCF-7 and ZR-751 cells show loss of ERα and PgR expression and gain in EGFR expression with a shift from ER to EGFR signalling pathways. **A** and **B** Expression profiles of signalling receptors, ERα, PgR, Her2 and EGFR in MCF-7 and MCF-7 RR cell lines using **A** IHC with MTS and **B** Western blot analysis. **Ci** SRB at 72 h and **Cii** scratch assay at 24 and 48 h showing the effects of the gefitinib in MCF-7 and RR cell lines. **Ciii** SRB assay showing the effect of tamoxifen in MCF-7 and RR cell lines at 72 h. **D** Levels of EGFR expression increased 96 and 120 h after ERα siRNA knockdown. Graphs document the relative intensity of EGFR normalised to the loading controls using 25 nM of ERα siRNA. **Ei&ii** SRB assay showing significant reduction in proliferation of the MCF-7 cells after 72 h treatment with either ERα siRNA (25 nM) or tamoxifen (0.05 μM), whereas no reduction in proliferation was seen in MCF-7 RR cells. **Fi&ii** SRB assay showing an additional reduction in MCF-7 proliferation following ERα knockdown or tamoxifen treatment when combined with radiation 24 h after transfection/tamoxifen treatment (2-way ANOVA with Holm-Sidak’s multiple comparisons test; data expressed as mean ± SEM, *n* = 3, ****p ≤ 0.0001; **p ≤ 0.01; *p ≤ 0.05)

Breast cancer subtypes can be characterised by the expression profiles of key signalling receptors (ERα, PgR, HER2 and EGFR). Western blotting and IHC showed that the RR phenotype in the cell lines derived from ER+ cells was characterised by the loss of ERα and PgR expression, along with a gain in total EGFR expression (Fig. 6A & B and Additional file 7: Figure S4 and Additional file 8: Figure S5). ICC with quantitative analysis confirmed reduction in the percentage of cells staining positive for ERα expression in the MCF-7 RR (0.27 ± 0.17%) cell line compared with the parental MCF-7 cell line (81 ± 1.1%) and in the ZR-751 RR (0.52 ± 0.36%) cell line compared with the parental ZR-751 (96.5 ± 1.8%) cell line. No Staining was seen in the MDA-MB-231 cell line or its RR derivative. Three replicates with a minimum of 10 spheroids were included in each analysis. To investigate the consequences of this gain in EGFR expression, we treated parental and RR cells with increasing doses of the EGFR inhibitor, gefitinib, and determined its effect on the proliferation and migration of ER+ parental and RR cell lines. There was a statistically significant decrease in MCF-7 RR proliferation after 72 h with gefitinib concentrations ranging from 0.1–15 μM compared to the parental cell lines (IC_50_: MCF-7 13.36 μM, MCF-7 RR 6.43 μM) and a significant reduction in migration, with a dose of 5 μM decreasing the migratory potential of the RR cell line to parental levels (Fig. 6Ci & Cii). Similar results were also seen in the ZR-751 parental and RR cell lines when treated with gefitinib (Additional file 9: Figure S6). As we had identified a loss in ERα expression, we also examined the effects of tamoxifen on the cell lines. After 72 h of tamoxifen treatment there was a statistically significant increase in proliferation of the MCF-7 RR cell line compared to the parental cell line at concentrations ranging from 0.01–3 μM (Fig. 6Ciii).

### ERα knockdown in MCF-7 cells results in EGFR expression, reduction in proliferation and enhancement of radiosensitivity

The loss of ERα in the MCF-7 cell line was further investigated using ERα siRNA knockdown. ERα knockdown was associated with increased EGFR expression 96 and 120 h after transfection, as shown through western blot analysis (Fig. 6D). There was also a significant reduction in proliferation of the MCF-7 cells after 72 h treatment with either ERα siRNA (25 nM) or tamoxifen (0.05 μM) (Fig. 6Ei), whereas no reduction in proliferation was seen in the MCF-7 RR cells (6Eii). An additional reduction in MCF-7 proliferation was observed when ERα knockdown or tamoxifen treatment was combined with radiation 24 h after transfection/tamoxifen treatment (Fig. 6Fi&ii), suggesting a radiosensitising rather than a radioprotective effect from ERα loss.

### Radioresistant cell lines exhibit cellular plasticity within the context of intrinsic breast cancer subtyping

**Fig. 7:**
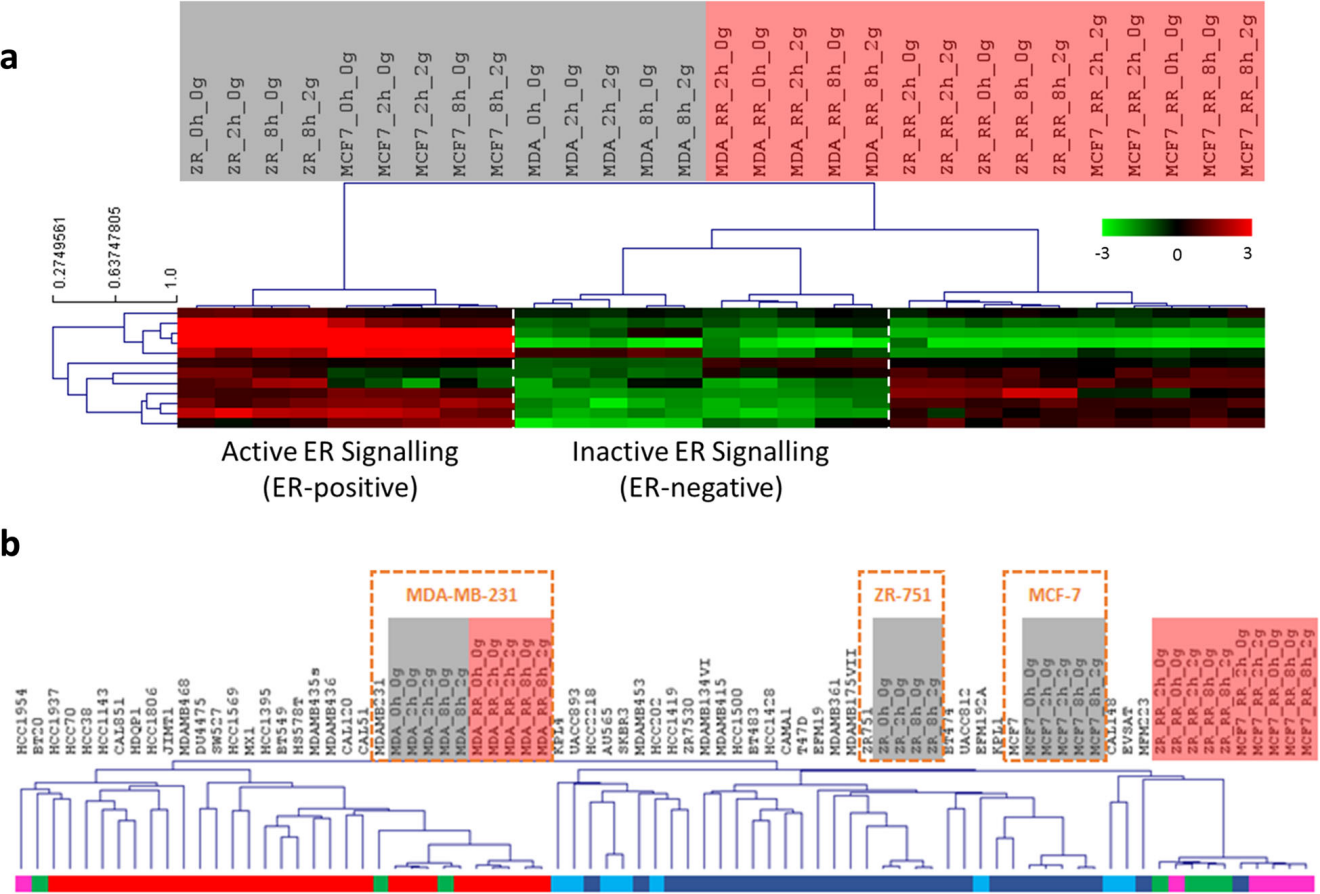
ER+ derived radioresistant cell lines exhibited a change in oestrogen signalling associated genes and a change in intrinsic breast cancer subtyping. **a** Heatmap showing log2 mean-centered expression profile of a published oestrogen-signalling gene signature [26]; red = higher expression, green = lower expression. Heatmap clustering was carried out using Pearson correlation with average linkage. **b** Study data was integrated with a public gene expression dataset (GSE50811) of 67 breast cancer cell lines. Hierarchical clustering of parental and RR cell lines based on Pearson correlation to centroids of Sørlie 2003 intrinsic genes [16]; Red = Basal, Dark blue = Luminal A, Light blue = Luminal B, Purple = HER2-overexpressing, Green = Normal-like

ER signalling was further investigated through the application of a published gene expression signature for ER pathway signalling activity to our transcriptomic data [26]. As expected, the ER+ parental cell lines (MCF-7 and ZR-751) were characterised by high expression of these genes whereas the ER- parental and RR cell lines (MDA-MB-231) had lower expression. Interestingly, the MCF-7 RR and ZR-751 RR cells clustered with the ER- cell lines in hierarchical clustering analysis, although they maintained higher expression levels, similar to their parental cells, for a subset of these genes. Treatment with 2 Gy of radiation for 2 and 8 h was not found to influence the expression of these genes (Fig. 7a).

Gene expression data was integrated with a public gene expression dataset (GSE50811) of 67 breast cancer cell lines. The MDA-MB-231 parental and RR cell lines, both treated and untreated, clustered tightly with each other in the dendrogram branch enriched for the basal breast cancer subtype. Both treated and untreated MCF-7 and ZR-75-1 parental cell lines were classified as luminal A whereas their RR derivates clustered independently and were enriched for normal-like (ZR-751 RR) and HER2-overexpressing (MCF-7 RR) intrinsic subtypes (Fig. 7b and Additional file 10: Figure S7).

### PI3K and MAPK activity are increased in ER+ derived radioresistant cell lines

**Fig. 8:**
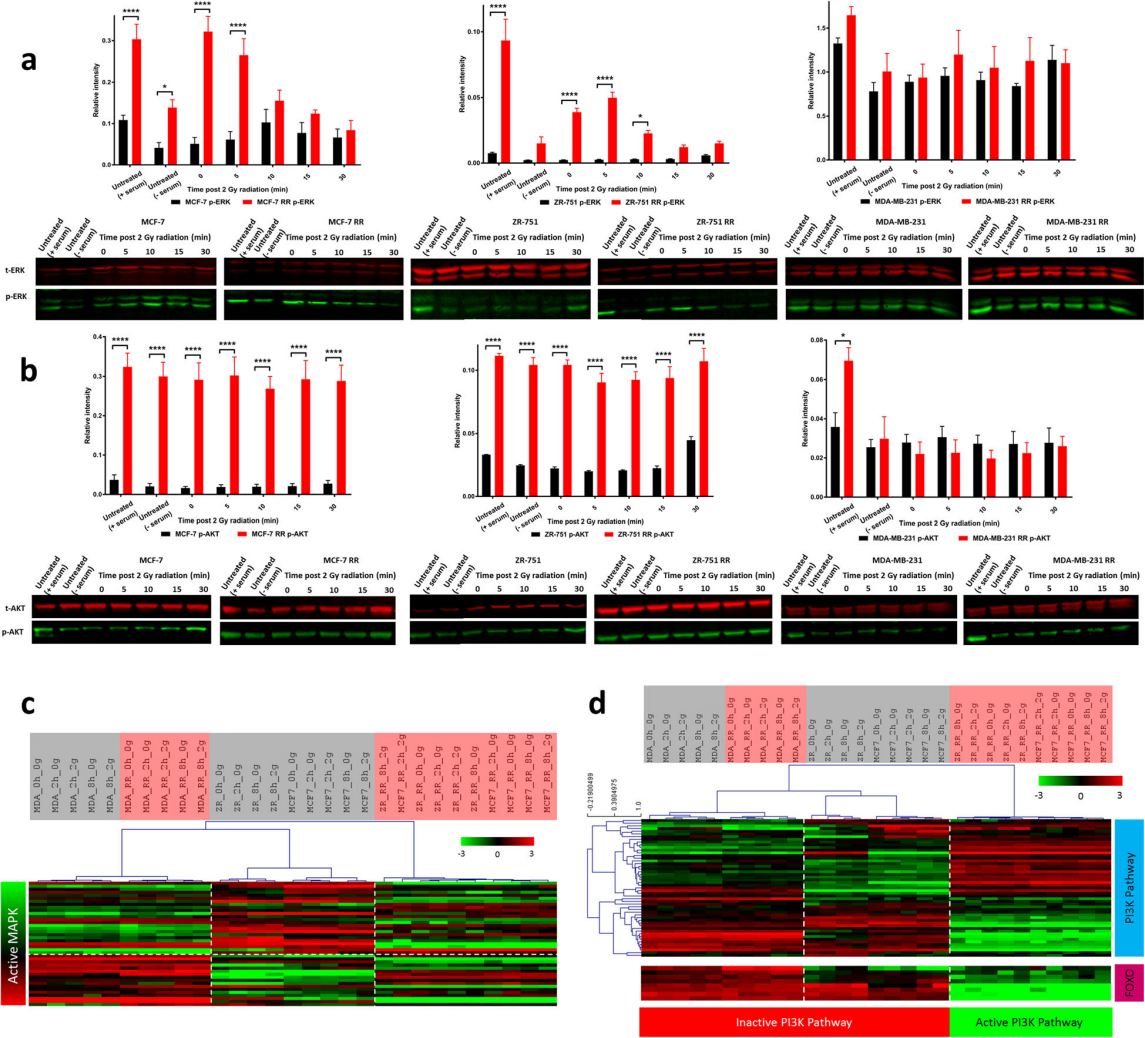
Activation of PI3K and MAPK pathways in ER+ derived radioresistant cell lines. **a** and **c** MAPK pathway activity; **b** and **d** PI3K pathway activity. **a** Western blot analysis showing the levels of p-ERK in response to a single fractionated dose of 2 Gy radiation in MCF-7, ZR-751 and MDA-MB-231 cell lines in comparison to their RR derivatives. **b** Western blot analysis showing the levels of p-AKT in response to a single fractionated dose of 2 Gy radiation in MCF-7, ZR-751 and MDA-MB-231 cell lines in comparison to their RR derivatives (2-way ANOVA with Holm-Sidak’s multiple comparisons test; data expressed as mean ± SEM, *n* = 3, ****p ≤ 0.0001; *p ≤ 0.05). **c** Heatmap showing log2 mean-centered gene expression profiles between parental and RR cell lines in respect to a MAPK pathway activity gene signature [27]. **d** Heatmap showing log2 mean-centered gene expression profiles between parental and RR cell lines in respect of the PI3K pathway (associated genes taken from the PI3K KEGG pathway (upper heatmap) and FOXO-regulated genes (lower heatmap) [28]. Heatmap clustering was carried out using Pearson correlation with average linkage

Downstream signal transduction pathways of the HER/ERBB tyrosine-kinase receptor family were further investigated following identification that total EGFR expression was increased in the ER+ derived RR cell lines. Western blot analysis of time course experiments up to 30 min after 2 Gy radiation showed increased levels of p-ERK in MCF-7 RR and ZR-751 RR cell lines 5 min post-radiation, whereas a smaller increase was seen in the parental cell lines at a later time point (Fig. 8a). Although p-AKT did not show increased levels in response to radiation, the RR cell lines had overall increased expression levels compared with the parental cell lines (Fig. 8b). No differences were seen in the MDA-MB-231 and RR cell lines. The activity of the MAPK pathway in our cell lines was investigated using a published gene expression signature [27]. PI3K activity was assessed using genes taken from the KEGG pathway database [25] in combination with FOXO-regulated genes (these have an inverse expression pattern to PI3K activity) [28]. Gene expression analysis suggested that active MAPK and inactive PI3K signalling were constitutive in the MDA-MB-231 parental and RR cell lines and expression levels of these signalling pathways were not affected by radiation treatment. The untreated ER+ cell lines were characterised by both inactive MAPK and PI3K activity, whereas their RR derivatives had lower overall expression of the MAPK-associated genes, potentially representing a switch to active MAPK and PI3K signalling (Fig. 8c and d).

## Discussion

Intrinsic or acquired radioresistance can be a significant obstacle in the treatment of many cancers, including breast. In this study we developed novel radioresistant cell lines from both ER+ and ER- breast cancer cell lines and characterised their inherent differences and responses to radiation through genetic, molecular and cell biology approaches. Radioresistance was acquired in all 3 RR cell lines, which validated their use as an in vitro model system to help characterise this phenotype and investigate the mechanisms involved in radioresistance development and treatment failures in patients. The radioresistant phenotype was also shown to be maintained in MCF-7 RR cells that were not exposed to radiation for 6 months indicating that the acquisition of radioresistance is not transient. This has important therapeutic implications and is in contrast to a previous study that showed radioresistant MCF-7 cells lost their resistant phenotype 12 weeks after a final dose of radiation [29]. The difference in maintenance of the RR phenotype could be due to our cell lines being exposed to radiation over a longer time period, which could contribute to more stable phenotypic changes and highlights the importance of performing regular CF/SRB assays to confirm the maintenance of radioresistance.

Only a small number of other studies have investigated mechanisms of acquired RR through the generation of RR cell lines, which tend to focus on a single specific pathway [7, 29–33]. Reduced proliferation rates have been identified in RR cell lines [34]; reduced proliferation in our MCF-7 RR and ZR-751 RR cell lines was reflected in the gene expression profiles and reduced Ki67 expression in MTS. One study used 3 prostate cancer cell lines (androgen responsive and non-responsive) to develop RR derivates; they identified enhanced EMT/cancer stem cell phenotypes and activation of checkpoint proteins and PI3K/AKT/mTOR signalling pathways in the RR cells [31]. Our study took a similar approach by using 3 breast cancer cell lines, 2 ER+ and hormone responsive (MCF-7 and ZR-751) and 1 ER- and hormone non-responsive (MDA-MB-231). However, our approach was more global and investigated multiple potential radioresistance mechanisms rather than focusing on individual pathways.

Among breast cancer patients, more than 90% of cancer related deaths are from metastatic rather than primary disease [35]; further understanding of breast cancer metastasis and treatment failure is therefore of clinical importance. We identified that the MCF-7 RR and ZR-751 RR cell lines had increased invasion and migration ability compared with their parental cell lines whereas the MDA-MB-231 RR cell line exhibited increased migration but had only a marginal, non-significant increase in invasion compared to the parental cells. The results suggest that these RR cell lines developed a more aggressive phenotype, which was more pronounced in the ER+ derived RR cell lines, which clinically could lead to a greater potential for metastasis.

To model this further we used well established MTS models that more accurately represent the tumour microenvironment within a solid tumour; MTS exhibit 3D cell-cell interactions and can develop O_2_, nutrient and catabolite gradients, producing a central necrotic core and outer proliferating layer of cells [36]. The novel MTS derived from RR cells developed in this study serve as excellent tumour models of radioresistance to investigate invasiveness and protein expression through IHC, which aided in their classification.

EMT is an important process in malignant transformation that leads to reduced cell–cell contacts, increased cell motility and metastasis [37–39]. Loss of epithelial morphology is an important prognostic indicator that correlates with worse prognosis [40]. Hybrid/intermediate phenotypes show the ability of cancer cells to undergo EMT and mesenchymal-to-epithelial-transition, thus traversing the epithelial and mesenchymal states [38]. In our RR model we demonstrated a down regulation of E-cadherin and upregulation of vimentin, N-cadherin and SNAIL, all known biomarkers for EMT in breast cancer [41], in the MCF-7 RR and ZR-751 RR cell lines. These results support the morphological and functional changes in RR cells, which developed a more mesenchymal-like phenotype with increased invasive and migratory potential. The MDA-MB-231 cell line has previously been characterised as having a mesenchymal-like phenotype with constitutively low E-cadherin and high vimentin expression, so any further development of EMT in its RR derivative was difficult to ascertain. Many signalling pathways can activate EMT in both normal and cancer cells, including receptor tyrosine kinase signalling, TGFβ, Wnt-β-catenin and Notch signalling [42, 43]; radiation alone can also induce EMT through the expression of TGFβ [44, 45]. Both EGFR downstream signalling pathways and Wnt signalling were found to be activated in our MCF-7 RR and ZR-751 RR cell lines suggesting possible mechanisms by which the cells underwent EMT. It is interesting to note that the same population of cells, predominantly those around the periphery of the MTS formed from ER+ RR cells, showed increased co-expression of vimentin, Snail, N-cadherin and WNT5a.

Breast cancer is routinely characterised immunohistochemically by the expression of receptors such as HER2, ERα, PgR and EGFR; gene expression profiling can also be used to identify molecular characteristics of breast cancer cells and classify them into 5 specific subtypes with distinct clinical outcomes: luminal A, luminal B, HER2-overexpressing, basal and normal-like tumours [46, 47]. These different breast cancer subtypes have also been shown to exhibit differential inherent sensitivities to ionising radiation and reflect prognosis [48, 49]. The clinical usefulness of molecular subtyping of cell lines has been previously investigated through comparing the genomic and transcriptional characteristics of breast cancer cell lines with that of primary breast tumours. One study showed that recurrent genome aberrations and the resulting transcriptional changes identified in 51 cell lines (including MDA-MB-231, MCF-7 and ZR-751) were well preserved in comparison to those found in primary tumours of a similar classification and that cell lines may not accumulate substantial new mutations during extended culture, showing stable genomic patterns over multiple passages [50]. Although the cell lines carried more genome aberrations, possibly related to some cell lines having been derived from late stage tumours or pleural effusions, they concluded that cell lines were well suited to assess the functional consequences of genome-aberrations-mediated gene deregulation and to identify molecular features that predict resistance/sensitivity to agents targeting these aberrations. However, it is important to note that cell line expression profiles will not account for the tumour microenvironment including normal epithelial cells or stromal tissue, nor will they reflect intra-tumoural heterogeneity. With these caveats in mind the purpose of subtyping in this study was to demonstrate that the development of radioresistance is consistent with a change to a prognostically less favourable intrinsic subtype.

In our study the transcriptional profiles of parental and RR cell lines were assigned to intrinsic breast cancer subtypes. Both the MDA-MB-231 parental and RR cell lines were triple negative, and subtyping identified them as basal [16, 46, 51, 52]. As the MDA-MB-231 parental cell line is inherently aggressive, it is not surprising that no significant changes were identified in its RR derivative. However, a change in classification was identified in RR cell lines derived from ER+ cells, with a shift from luminal A for both MCF-7 and ZR-751 cell lines towards a non-luminal classification. The MCF-7 RR cell line was most closely correlated to the HER2-overexpressing subtype while the ZR-751 RR cell line was more closely correlated to the normal-like subtype. Both HER2-overexpressing and normal-like subtypes carry a worse prognosis compared to luminal A tumours, with HER2-overexpressing tumours having a higher risk of locoregional recurrence [16, 46, 51, 52]. Luminal A tumours also respond well to hormone and radiation treatment [51], and a shift away from this subtype would therefore be consistent with a loss of radiosensitivity, resistance to endocrine therapy and a more aggressive phenotype. These results show that the acquisition of radioresistance can be linked with cellular plasticity through extensive alterations in gene expression resulting in a change in molecular subtype.

Analysis of protein expression indicated that both MCF-7 RR and ZR-751 RR cell lines lost both ERα and PgR expression and gained EGFR expression. Furthermore, ERα siRNA knockdown resulted in increased EGFR protein expression in the MCF-7 cell line, suggesting a direct relationship between loss of ERα and increased EGFR expression. Using gene analysis, we further identified that ER driven genes had lower expression in the RR cell lines derived from ER+ cell lines. An inverse relationship between ER activity and EGFR and HER2 expression has been reported in clinical breast cancer, with overexpression of these receptor tyrosine kinases being associated with decreased sensitivity to endocrine therapy and a poorer prognosis [53–55]. An apparent switch from ER signalling to EGFR-mediated signalling was investigated by the treatment of MCF-7 and MCF-7 RR cells with the EGFR inhibitor gefitinib and the anti-oestrogen tamoxifen.

Gefitinib produced a greater concentration-dependent reduction in cell proliferation in the MCF-7 RR cell line with an IC_50_ value of 6.43 μM. This is a relatively high concentration compared to clinically relevant doses [56–58]; however, while this suggests the MCF-7 RR cell line is relatively insensitive to the effects of gefitinib, there was a significant reduction in the IC_50_ value from 13.34 μM for the MCF-7 parental cell line. Results also showed that the RR cells developed simultaneous resistance to tamoxifen at clinically relevant doses. Tamoxifen may be given to patients with ER+ breast cancer for 3–5 years following surgery and radiotherapy; if radioresistant cells remain following a patient’s RT course then the efficacy of tamoxifen in radioresistant cells needs to be assessed. Mechanisms for acquired tamoxifen resistance are complex since tamoxifen resistant tumours usually do not lose ERα [59], with the receptor still remaining functional as demonstrated by cohorts of patients with recurrent disease still being able to respond to secondary endocrine therapy [60, 61]. This has led to studies suggesting that tamoxifen resistance is through modification of ER functionality by growth factor pathways [62, 63]. A previous study using MCF-7 RR cells demonstrated resistance to tamoxifen without a change in ER expression. However, AKT phosphorylation was increased and the investigators suggested that sequential tamoxifen treatment following radiation could be more effective than concurrent treatment [33]. Conversely, results from our study showed that when radiation was delivered 24 h after tamoxifen treatment or ERα knockdown in the MCF-7 cells, an additional decrease in proliferation was observed, suggesting that the use of tamoxifen before and during a patient’s RT treatment may be advantageous. Another study determined that tamoxifen-resistant MCF-7 cells, showed enhanced sensitivity to EGF and oestradiol stimulation. Although ERα was not lost, it was found to redistribute to extra-nuclear sites (cytoplasmic translocation) in the tamoxifen-resistant cells and they suggested that enhanced ERα function, via cooperation with EGFR, is one mechanism responsible for acquired tamoxifen resistance [64]. Similar results have also been shown with the establishment of anti-oestrogen-resistant MCF-7 cells, developed through continuous culture in fulvestrant (trade name Faslodex). These resistant cells developed reduced ERα expression and loss of PgR with increased dependence on EGFR/MAPK mediated signalling [65]. Our radioresistant model has shown that ERα and PgR loss occurs with concurrent resistance to tamoxifen which may involve different pathways compared to the development of tamoxifen resistance from chronic drug exposure. This suggests that additional targeted therapy may be required in these cells.

Because total EGFR expression was significantly increased in the RR cell lines, the downstream signal transduction pathways of the HER tyrosine-kinase receptor family were further investigated.

The phosphatidylinositol-3-kinase (PI3-k)/protein kinase B (AKT) cascade is frequently overactive in a wide range of cancers and can trigger a cascade of responses related to cell survival and metabolism [66, 67]. It is also activated by RT and has been associated with resistance mechanisms such as intrinsic radioresistance, tumour cell proliferation and hypoxia [68, 69]. In this study we found that the gene expression signature in the MCF-7 RR and ZR-751 RR cell lines fitted with activation of the PI3K pathway. Although p-AKT did not increase in response to radiation, its levels were statistically higher than in the parental derivatives, suggesting that the PI3K/AKT/mTOR signalling pathway is activated in these RR cell lines and may play an important role in radioresistance.

In breast and other cancers radiation-induced proliferation has been linked with activation of EGFR and downstream components of the mitogen-activated protein kinase (MAPK) cascade, including phospholipase-C, Ras, and Raf-1 [70–73] and can be considered an important cytoprotective response [74]. MAKP activation may represent a mechanism by which cancer cells can cause tumour repopulation during fractionated RT courses. Results from this study at the gene and protein level suggest that MAPK signalling is activated in the MCF-7 RR and ZR-751 RR cell lines since basal levels of p-ERK1 and p-ERK2 were significantly higher than in their parental cell lines and phosphorylation of ERK1 and ERK2 occurred almost immediately following a 2 Gy radiation dose. This radiation-induced phosphorylation occurred more quickly and to a larger extent than in the parental cell lines. Our results suggest that EGFR signalling, and multiple components of its downstream pathways are activated in the MCF-7 RR and ZR-751 RR cell lines, which could have significant clinical implications, as multiple pathways may need to be targeted to improve the therapeutic response to irradiation.

The use of multiple techniques ranging from gene to protein to functional experiments, which led to the validation of our results through independent means, represents one of the main strengths of this study. Additionally, the development of ER+ and ER- RR cell lines allowed for comparisons to be made between different molecular breast cancer subtypes. The aim of our study was to characterise these RR cell lines and identify pathways that could be related to radioresistance, rather than focusing on the reversal of radioresistance itself. However, following our successful characterisation, we now aim to use these cell lines in future studies to target these identified pathways and to identify biomarkers (genetic and secretomic signatures) that could be used for determining radiosensitivity and for the assessment of a tumour’s response to radiotherapy.

## Conclusion

In this study we developed a novel model to help improve the understanding and characterisation of radioresistant breast cancer cells and identified several important and likely interrelated networks contributing to the development of radioresistance. ER+ derived RR cell lines were characterised by a shift towards a more invasive mesenchymal phenotype with changes in oestrogen regulation, gain of EGFR signalling and change in subtype classification. The ER- cell line possessed these characteristics at the outset and hence its phenotype changed relatively little with the development of radioresistance. Here we have begun to dissect the mechanisms and signalling pathways involved in the development of radioresistance. Models of resistant disease as developed and examined in this study will be instrumental in future research and will aid the development of new therapeutic strategies for patients that either fail to respond or develop recurrent disease.

## Additional files

Additional file 1: **Table S1.** RIN values for all samples used in gene expression analysis. (XLSX 10 kb)

Additional file 2: **Table S2.** Overview of samples (cell line, treatments, time points, replicates) used in each experiment or analysis. (XLSX 15 kb)

Additional file 3: **Figure S1.** Colony formation assay comparing MCF-7, MCF-7 RR and MCF-7 rr (radioresistant cell line not radiated for 6 months (24 passages)) cell lines. (TIF 51 kb)

Additional file 4: **Figure S2.** ICC and IHC staining of EMT markers in ZR-751 parental and RR cell lines. (TIF 2384 kb)

Additional file 5: **Figure S3.** ICC and IHC staining of EMT markers in MCF-7 parental and RR cell lines. (TIF 2391 kb)

Additional file 6: **Table S3.** Gene lists from a published EMT signature [23] and WNT signalling associated genes [25] applied to our gene expression data. (XLS 62 kb)

Additional file 7: **Figure S4.** ICC and IHC staining for signalling receptors in ZR-751 parental and RR cell lines. (TIF 2818 kb)

Additional file 8: **Figure S5.** ICC and IHC staining for signalling receptors in MCF-7 parental and RR cell lines. (TIF 2708 kb)

Additional file 9: **Figure S6.** (A) SRB at 72 h and (B) Scratch assay at 24 h showing the effects of gefitinib on ZR-751 and ZR-751 RR cell lines (2-way ANOVA with Holm-Sidak’s multiple comparisons test; data expressed as mean ± SEM, *n*=3, ****p≤0.0001; ***p≤0.001; **p≤0.01; *p≤0.05). (TIF 170 kb)

Additional file 10: **Figure S7.** Gene expression heatmap based on Pearson correlation hierarchical clustering with average linkage using a published list of intrinsic subtype genes [16]. Intrinsic subtype was assigned using the *genefu* R package Single Sample Predictor (SSP) algorithm [17]; red=higher expression, green=lower expression. Red=Basal, Dark blue=Luminal A, Light blue=Luminal B, Purple=HER2-overexpressing, Green=Normal-like. (TIF 758 kb)

## Abbreviations

ATP: Adenosine triphosphate
BCA: Bicinchoninic acid
BCS: Breast conserving surgery
CF: Colony formation
DMEM: Dulbecco’s modified Eagle’s medium
DNA: Deoxyribonucleic acid
DSB: Double strand break
EGFR: Epidermal growth factor receptor
EMT: Epithelial-to-mesenchymal transition
ER: Oestrogen receptor
ERK1/2: Extracellular-signal regulated kinases 1 and 2
FCS: Foetal calf serum
HER2: Human epidermal growth factor receptor 2
ICC: Immunocytochemistry
IHC: Immunohistochemistry
KEGG: Kyoto Encyclopaedia of Genes and Genomes
MAPK: Mitogen-activated protein kinase
MTS: Multicellular tumour spheroid
NF-ƙB: Nuclear factor kappa light chain enhancer of activated B cells
PgR: Progesterone receptor
PI3K: Phosphatidylinositol-4,5-bisphosphate 3-kinase
RNA: Ribonucleic acid
RR: Radioresistant
RT: Radiotherapy
RTK: Receptor tyrosine kinase
SRB: Sulforhodamine B
TGFβ: Transforming growth factor beta
WNT: Wingless-related integration site
