# Supplementary figures and images for: Development and characterisation of acquired radioresistant breast cancer cell lines
Source: Radiat Oncol. 2019 Apr 15;14:64. doi: 10.1186/s13014-019-1268-2 (PMC6466735; doi:10.1186/s13014-019-1268-2)

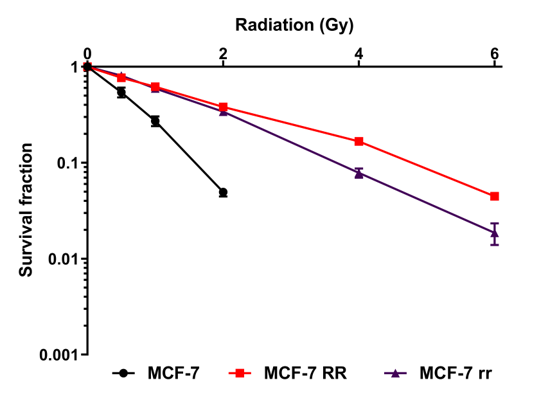

Supplement: Supplementary file 3 — Figure S1. Colony formation assay comparing MCF-7, MCF-7 RR and MCF-7 rr (radioresistant cell line not radiated for 6 months (24 passages)) cell lines. (TIF 51 kb) [file 13014_2019_1268_MOESM3_ESM.tif]

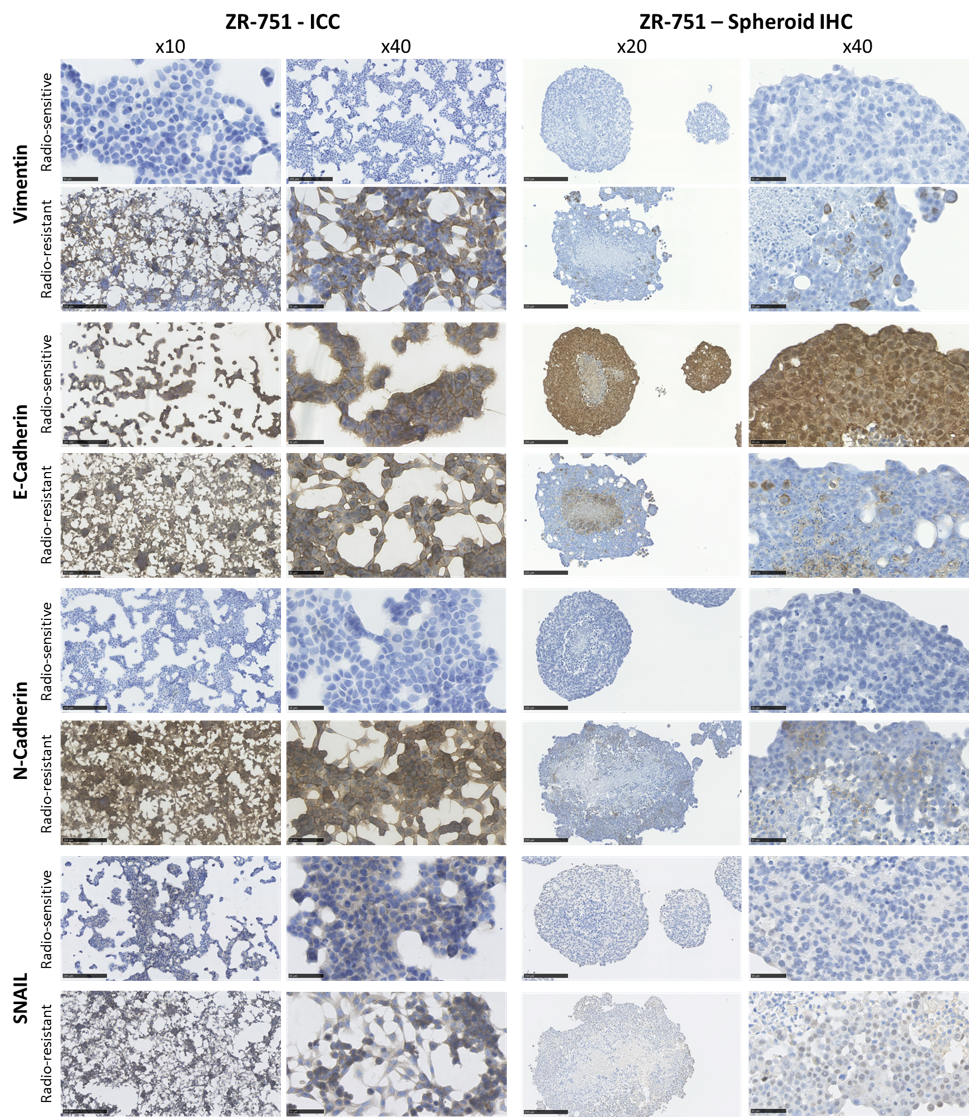

Supplement: Supplementary file 4 — Figure S2. ICC and IHC staining of EMT markers in ZR-751 parental and RR cell lines. (TIF 2384 kb) [file 13014_2019_1268_MOESM4_ESM.tif]

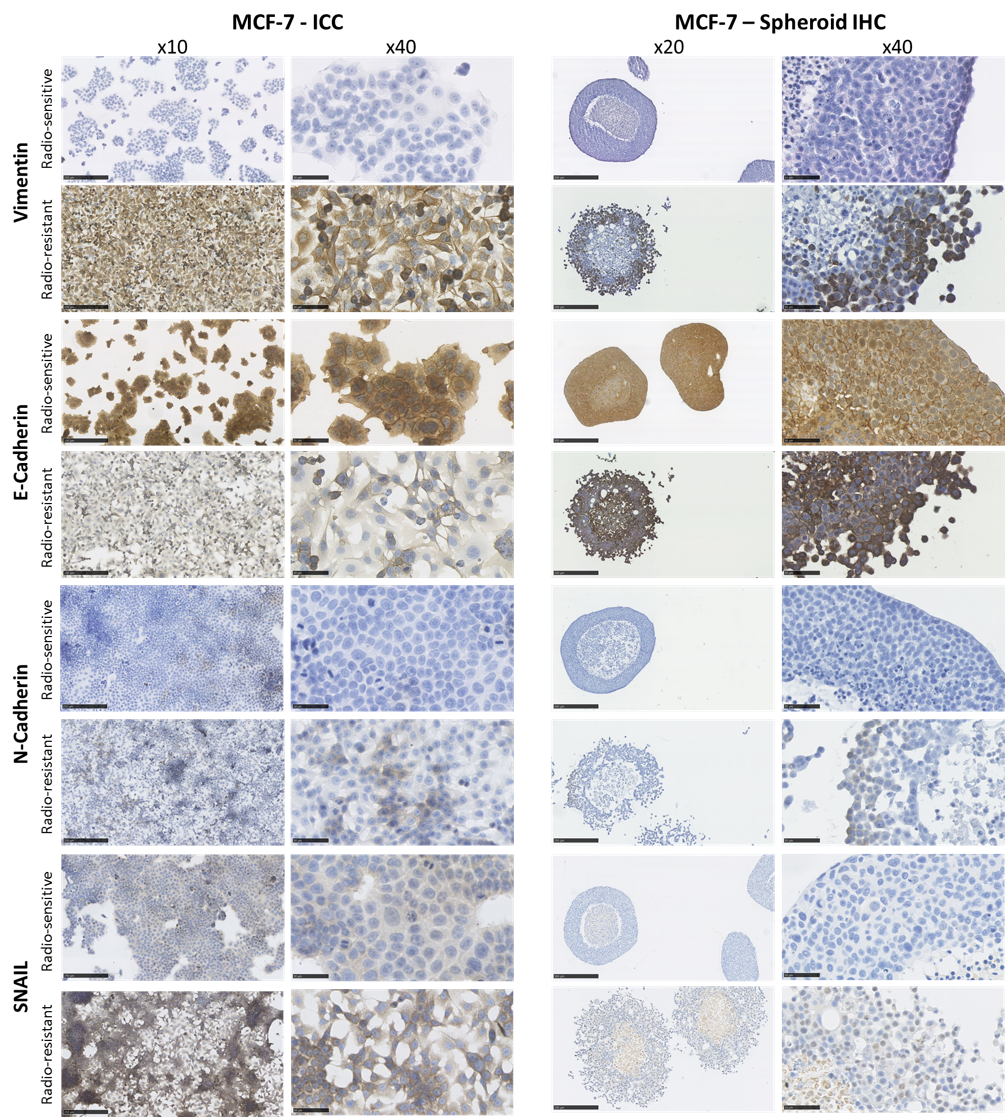

Supplement: Supplementary file 5 — Figure S3. ICC and IHC staining of EMT markers in MCF-7 parental and RR cell lines. (TIF 2391 kb) [file 13014_2019_1268_MOESM5_ESM.tif]

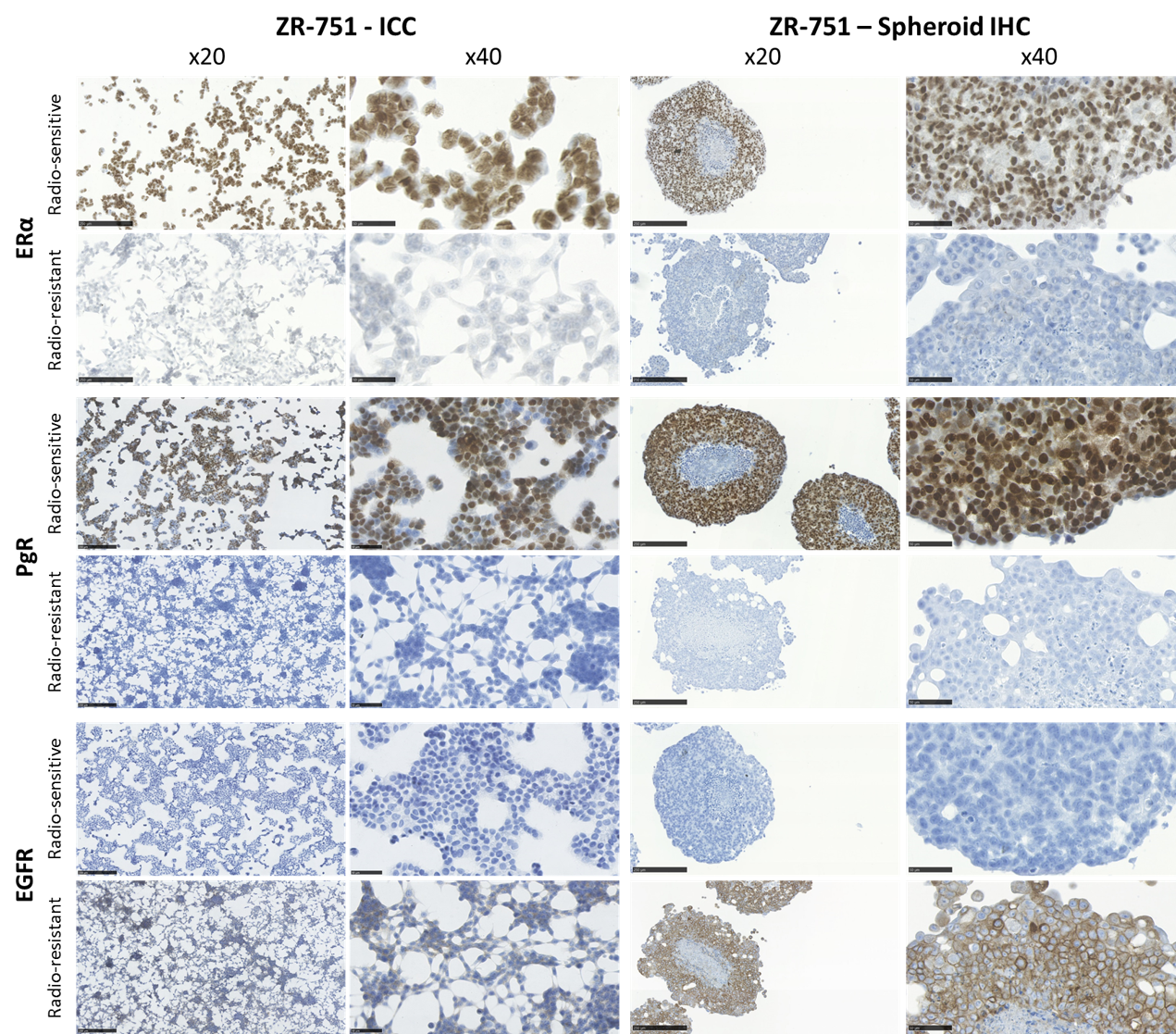

Supplement: Supplementary file 7 — Figure S4. ICC and IHC staining for signalling receptors in ZR-751 parental and RR cell lines. (TIF 2818 kb) [file 13014_2019_1268_MOESM7_ESM.tif]

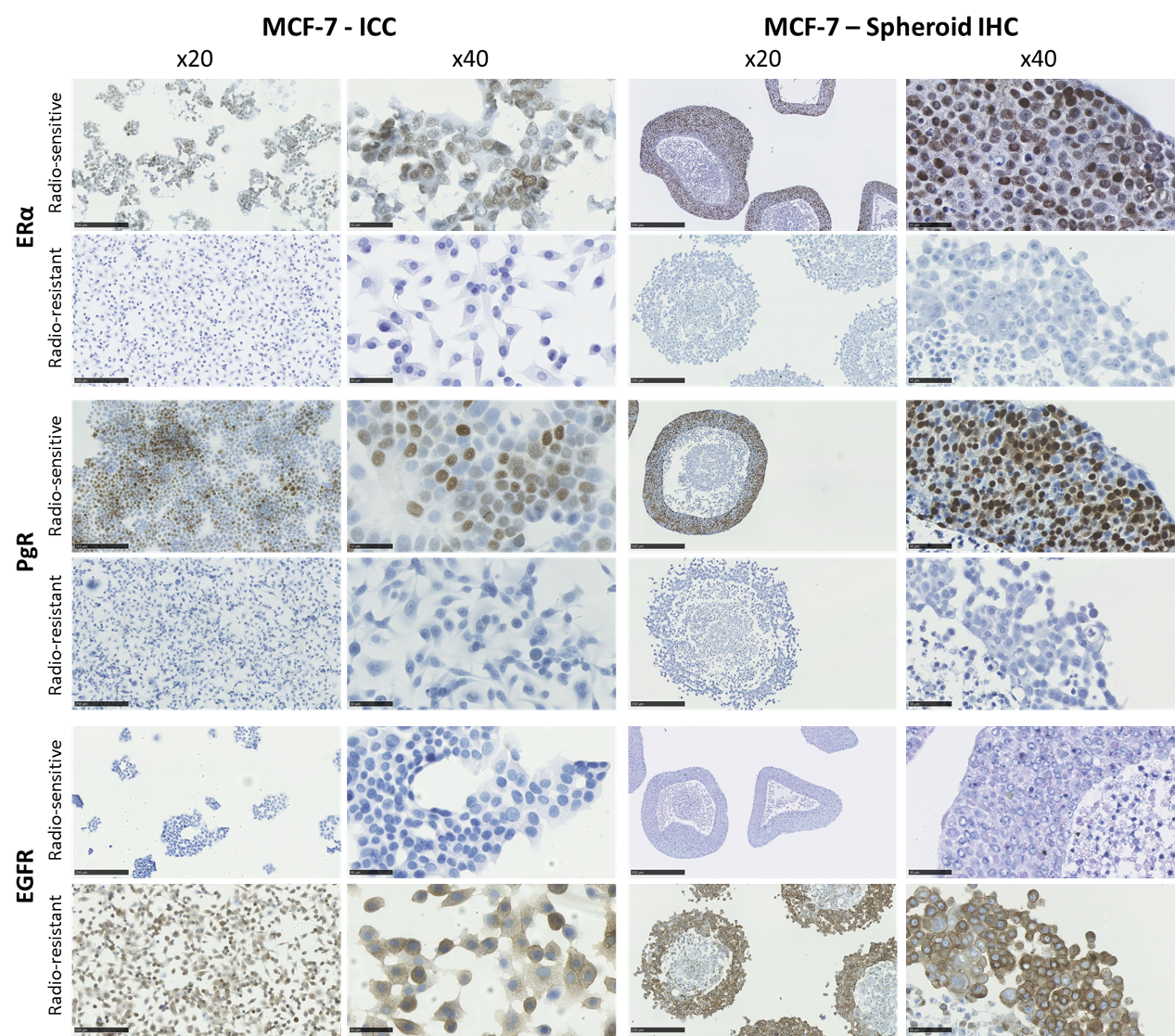

Supplement: Supplementary file 8 — Figure S5. ICC and IHC staining for signalling receptors in MCF-7 parental and RR cell lines. (TIF 2708 kb) [file 13014_2019_1268_MOESM8_ESM.tif]

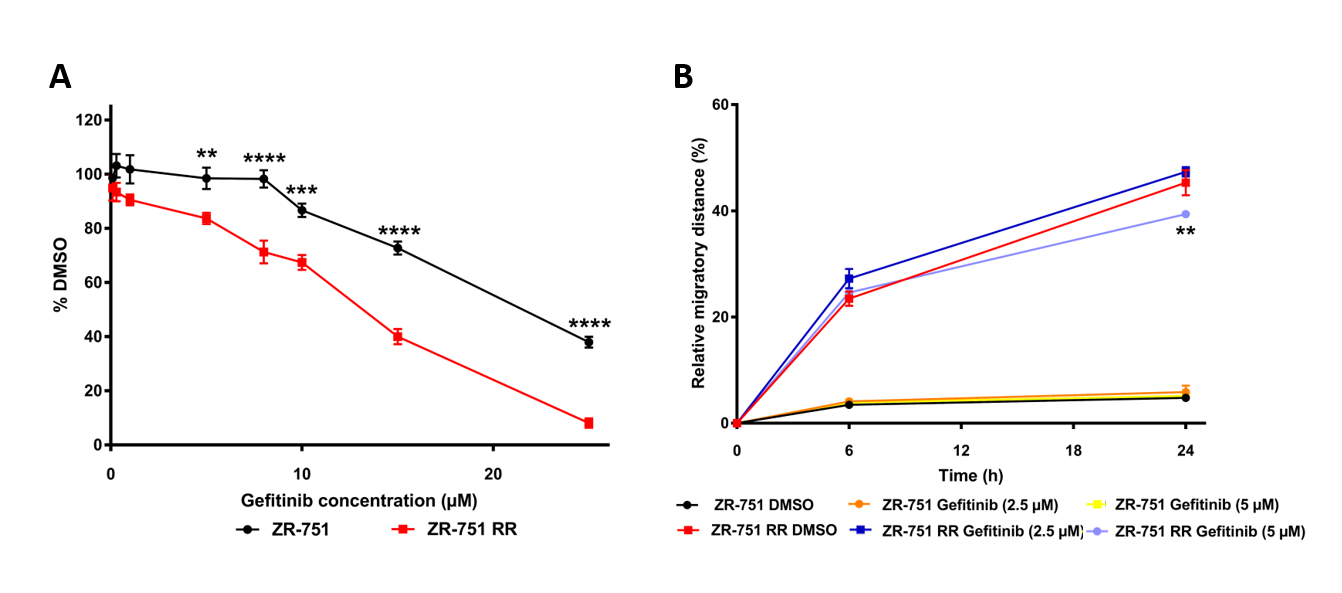

Supplement: Supplementary file 9 — Figure S6. (A) SRB at 72 h and (B) Scratch assay at 24 h showing the effects of gefitinib on ZR-751 and ZR-751 RR cell lines (2-way ANOVA with Holm-Sidak’s multiple comparisons test; data expressed as mean ± SEM, n=3, ****p≤0.0001; ***p≤0.001; **p≤0.01; *p≤0.05). (TIF 170 kb) [file 13014_2019_1268_MOESM9_ESM.tif]

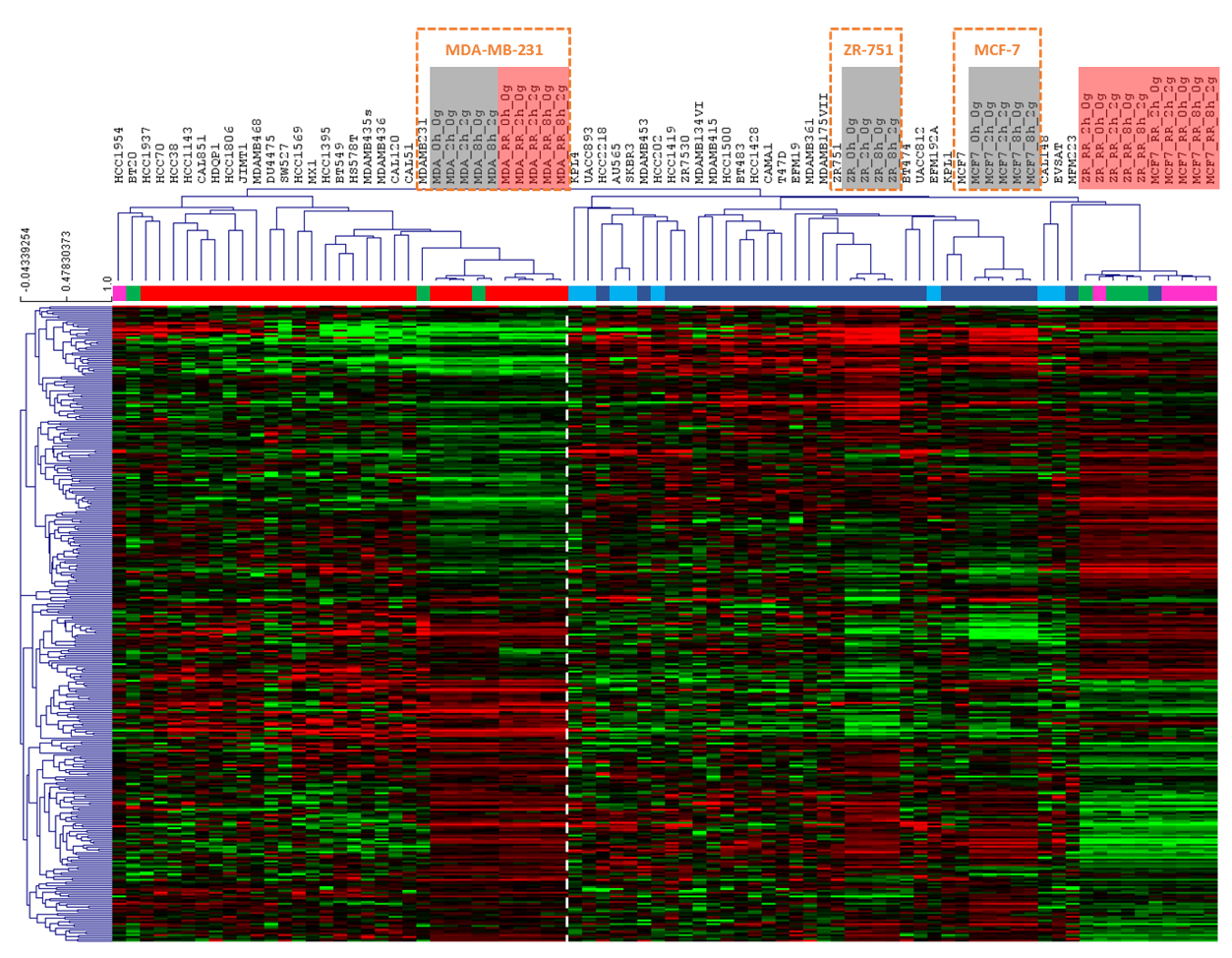

Supplement: Supplementary file 10 — Figure S7. Gene expression heatmap based on Pearson correlation hierarchical clustering with average linkage using a published list of intrinsic subtype genes [16]. Intrinsic subtype was assigned using the genefu R package Single Sample Predictor (SSP) algorithm [17]; red=higher expression, green=lower expression. Red=Basal, Dark blue=Luminal A, Light blue=Luminal B, Purple=HER2-overexpressing, Green=Normal-like. (TIF 758 kb) [file 13014_2019_1268_MOESM10_ESM.tif]
